# STAT3 Represses Nitric Oxide Synthesis in Human Macrophages upon *Mycobacterium tuberculosis* Infection

**DOI:** 10.1038/srep29297

**Published:** 2016-07-07

**Authors:** Christophe J. Queval, Ok-Ryul Song, Nathalie Deboosère, Vincent Delorme, Anne-Sophie Debrie, Raffaella Iantomasi, Romain Veyron-Churlet, Samuel Jouny, Keely Redhage, Gaspard Deloison, Alain Baulard, Mathias Chamaillard, Camille Locht, Priscille Brodin

**Affiliations:** 1 University of Lille, CNRS, Inserm, CHU Lille, Institut Pasteur de Lille, U1019 – UMR 8204-CIIL-Center for Infection and Immunity of Lille, F-59000 Lille, France

## Abstract

*Mycobacterium tuberculosis* is a successful intracellular pathogen. Numerous host innate immune responses signaling pathways are induced upon mycobacterium invasion, however their impact on *M. tuberculosis* replication is not fully understood. Here we reinvestigate the role of STAT3 specifically inside human macrophages shortly after *M. tuberculosis* uptake. We first show that STAT3 activation is mediated by IL-10 and occurs in *M. tuberculosis* infected cells as well as in bystander non-colonized cells. STAT3 activation results in the inhibition of IL-6, TNF-α, IFN-γ and MIP-1β. We further demonstrate that STAT3 represses iNOS expression and NO synthesis. Accordingly, the inhibition of STAT3 is detrimental for *M. tuberculosis* intracellular replication. Our study thus points out STAT3 as a key host factor for *M. tuberculosis* intracellular establishment in the early stages of macrophage infection.

Tuberculosis (TB) is caused by *Mycobacterium tuberculosis,* a slow-replicating intracellular bacterium. Despite the availability of the BCG vaccine and multi-drug therapies, TB remains one of the most widespread bacterial infections in the world responsible for 1.4 million deaths annually and an increasing number of drug-resistant cases are reported each year^1^.

Primary infection occurs through inhalation of aerosol droplets harboring the tubercle bacilli. Once in the lungs, the alveolar macrophages are the main reservoir of the mycobacteria. Following uptake by the macrophage, *M. tuberculosis* replicates within this professional phagocytic cell using a variety of defense mechanisms^2^,^3^. A host-pathogen cross-talk is established, thereby, creating a favorable niche for intracellular survival of *M. tuberculosis* for prolonged periods of time in its human host. *M. tuberculosis* affects the host’s immune responses through modulation of the cytokine environment. Innate immune responses were shown to play a major role in the outcome of mycobacterial infection and in the regulation of adaptive immune responses^4^. The progressive establishment of the adaptive immune response during *M. tuberculosis* infection leads to the aggregation of immune cells, forming an organized structure or granuloma^5^. The tight regulation of the immune balance, shaped by both mycobacteria and host cells, has a critical impact on the outcome of the granuloma. Eventually, two opposite fates are possible, *i.e.* bacterial containment or systemic dissemination^6^,^7^.

Transcriptomic analyzes carried out on organs from *M. tuberculosis* infected animals showed that the Signal Transducers and Activators of Transcription (STAT) activation network is tightly regulated in response to *M. tuberculosis* infection^8^,^9^. At the molecular level, STAT proteins mediate responses to a number of cytokines or to type I-IFN^10^,^11^,^12^. In mammals, the STAT family protein consists of seven members (STAT1, STAT2, STAT3, STAT4, STAT5A, STAT5B and STAT6) sharing a common structure composed of six functionally conserved domains^13^,^14^. Typically, the binding of cytokines onto their receptor leads to the activation and dimerization of STATs. Then, activated STATs translocate into the nucleus where they initiate transcription of target genes such as IFN stimulated genes (*ISGs*)^14^. STAT proteins are known to modulate expression of cytokines and their contribution in the outcome of infection with *M. tuberculosis* was previously reported^8^,^9^,^15^. STAT1 and STAT4 deficiencies were associated with a reduced immunity to mycobacteria characterized by a defective Interferon-γ (IFN-γ) response, which is essential for host protection^16^,^17^,^18^.

Concerning STAT3, this transcription factor was shown to be activated in response to multiple cytokines such as IL-6, IL-10 or G-CSF thus conferring to STAT3 the ability to regulate multiple cell functions even within the same cell type^19^. In TB immunity, the pleiotropic function of STAT3 plays a major role in the activation of anti-inflammatory program in myeloid cells as well as in the differentiation and activation of T cells, resulting in the control of *M. tuberculosis* infection^19^. While the role of STAT3 was established in TB immune response, there is little information regarding its implication in the intracellular adaptation of the bacteria during the first hours following human macrophage infection. To address this question, we investigated the role of STAT3 signaling within human primary macrophages during the 24 first hours of infection with *M. tuberculosis*. Using fluorescence microscopy, we observed that STAT3 was quickly activated following *M. tuberculosis* uptake. Strikingly, STAT3 activation also occurred in bystander macrophages lacking intracellular *M. tuberculosis*. We showed that the rapid STAT3 activation is initiated by IL-10 signaling. We further identified that STAT3 repressed the early production of inflammatory cytokines such as IL-6, TNF-α and IFN-γ as well as NO production. The control of early cellular events, operated by STAT3, thus creates a favorable niche for *M. tuberculosis* colonization and propagation.

## Results

### *M. tuberculosis* induces early activation of STAT3 signaling in both infected and non-infected bystander macrophages

**Figure 1:**
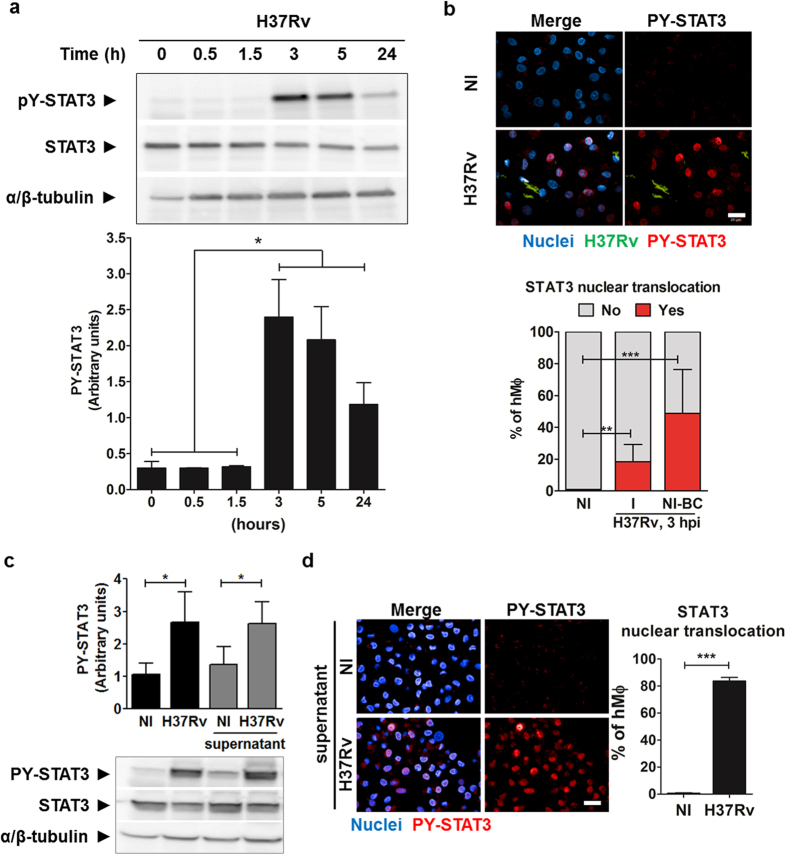
*M. tuberculosis* induces early activation of STAT3 signaling in both infected and bystander macrophages. (**a**) Kinetics of STAT3 activation in hMΦ infected with H37Rv at a MOI of 2. STAT3 activation was analyzed by immunoblotting using anti-PY^705^-STAT3 antibody. Immunoblot is representative of two independent experiments with two different donors. (**b**) Typical confocal images and image-based quantification of nuclear translocation of PY-STAT3 monitored by indirect immunofluorescence. Scale bar: 20 μm. Macrophage nuclei were stained using DAPI (blue), H37Rv-GFP are visualized in green and phosphorylated form of STAT3 was detected using antibody recognizing PY^705^-STAT3 (in red). Reported values represent the mean ± SD of the percentage of cells presenting STAT3 nuclear translocation from two independent experiments with two different donors (NI: Non infected, I: H37Rv-GFP infected cell, NI-BC: non infected –bystander cells). (**c**) Supernatants from H37Rv-GFP-infected hMΦ (H37Rv) and non-infected hMΦ (NI) were incubated with non-stimulated hMΦ. STAT3 activation was analyzed by immunoblotting using anti-PY^705^-STAT3 antibody. Immunoblots are representative of three independent experiments with three different donors. (**d**) Typical confocal images and image-based quantification of PY-STAT3 nuclear translocation monitored by indirect immunofluorescence. hMΦ were incubated for 3 h with filtered supernatant from uninfected hMΦ (NI) or supernatant from infected hMΦ (H37Rv). Macrophage nuclei were stained using DAPI (blue) and phosphorylated form of STAT3 was detected using anti-PY^705^-STAT3 antibody (red). Micrographs are representative of four independent experiments with four different donors. Scale bar: 20 μm. For each condition, about 1000 cells were analyzed, and the average percentages of cells presenting STAT3 nuclear translocation ± SD were plotted. For all immunoblots, Blotting with anti-STAT3 and anti-α/β-tubulin antibodies were used to confirm gel loading. Reported values represent the means ± SEM and correspond to the relative STAT3 phosphorylation. Full-length blots are presented in Supplementary Fig. S11. Asterisks indicate the statistically significant differences between compared conditions, calculated using the Student t-test (*p < 0.05, **p < 0.01, ***p < 0.001).

To investigate the effect of *M. tuberculosis* macrophages infection on the early phosphorylation of STAT3, primary human macrophages (hMΦ) were infected with *M. tuberculosis* H37Rv at a multiplicity of infection (MOI) of 2. We quantified the kinetics of the phosphorylation of STAT3 (PY-STAT3) by western blot, using specific antibodies directed towards Tyrosine 705. STAT3 was found to be highly phosphorylated 3 hours post-infection and the level of its phosphorylation was significantly maintained for 24 hours (Fig. 1a). Interestingly, a similar PY-STAT3 level was observed in macrophages incubated with killed H37Rv, showing that STAT3 signaling does not require interactions with live mycobacteria (Supplementary Fig. S1a).

Next, we studied the nuclear translocation of PY-STAT3 upon *M. tuberculosis* infection. To this end, hMΦ were infected with GFP-expressing *M. tuberculosis* H37Rv (H37Rv-GFP) for 3 hours and then labelled with DAPI nuclear stain. Confocal images were acquired on an automated microscope and image analysis was subsequently performed (Supplementary Fig. S2, Table S1). Using built-in image-analysis algorithms from Columbus (Perkin Elmer), cell borders could be defined using the DAPI image. A cell having overlapping green pixels that corresponds to H37Rv-GFP signal was then considered as a *M. tuberculosis* infected cell. Image-based quantification revealed that around 30% of cells were infected (Supplementary Fig. S1b,c). Consequently, around 70% of cells were considered non-infected bystander cells (NI-BC). Within these two populations- namely infected cells and NI-BC, we quantified the amount of cells presenting PY-STAT3 (Fig. 1b) nuclear translocation. We found that 20% of infected macrophages presented PY-STAT3 nuclear translocation. Interestingly, PY-STAT3 nuclear translocation also occurred in NI-BC, with 50% of nuclei positive for PY-STAT3 labeling (Fig. 1b). The activation of STAT3 signaling in NI-BC thus suggests that cell to cell communication was initiated after *M. tuberculosis* uptake by macrophages and could be quantified as early as three hours post-infection.

To better characterize the intercellular communication leading to a higher STAT3 activation in NI-BC, we analyzed its phosphorylation in naïve hMΦ stimulated with supernatant recovered from *M. tuberculosis-*infected macrophages. STAT3 was activated in macrophages stimulated with filtered supernatant and the level of phosphorylation was similar to that obtained from *M. tuberculosis* infected macrophages (Fig. 1c). Using immunofluorescence, PY-STAT3 showed a significant increase of nuclear translocation in hMΦ stimulated with filtered supernatant obtained from hMΦ infected with H37Rv-GFP (MOI 2, 3 h), as compared to hMΦ incubated with supernatant from non-infected cells (Fig. 1d). Approximately 80% hMΦ displayed STAT3 nuclear translocation following 1.5 hour of stimulation with filtered supernatant from *M. tuberculosis* infected samples. As control, no STAT3 translocation was found for supernatant from non-infected cells (Fig. 1d). These results indicate that STAT3 is quickly activated upon *M. tuberculosis* infection, suggesting a functional role for it in establishing an early immune response to *M. tuberculosis*.

### Activation of STAT3 mainly occurs through IL-10 signaling

Since STAT family members are transcription factors involved in cytokine signaling, we reasoned that cytokines might be secreted by *M. tuberculosis*-infected macrophages and trigger STAT activation. To identify the cytokines involved in STAT activation in our macrophage model system, we characterized the early release (5 hours post infection.) of 30 interleukines (IL) and chemokines by hMΦ in response to *M. tuberculosis* infection (Supplementary Fig. S3). *M. tuberculosis* phagocytosis strongly induced the secretion of chemotactic cytokines, such as monocyte chemotactic protein-1 (MCP-1/CCL-2), macrophage inflammatory protein-1α (MIP-1α/CCL-3) and 1β (MIP-1β/CCL-4) as well as IL-8 (CXCL-8). *M. tuberculosis* uptake also induced the release of inflammatory cytokines such as IL-1β, IL-12 and TNF-α (Supplementary Fig. S3).

**Figure 2:**
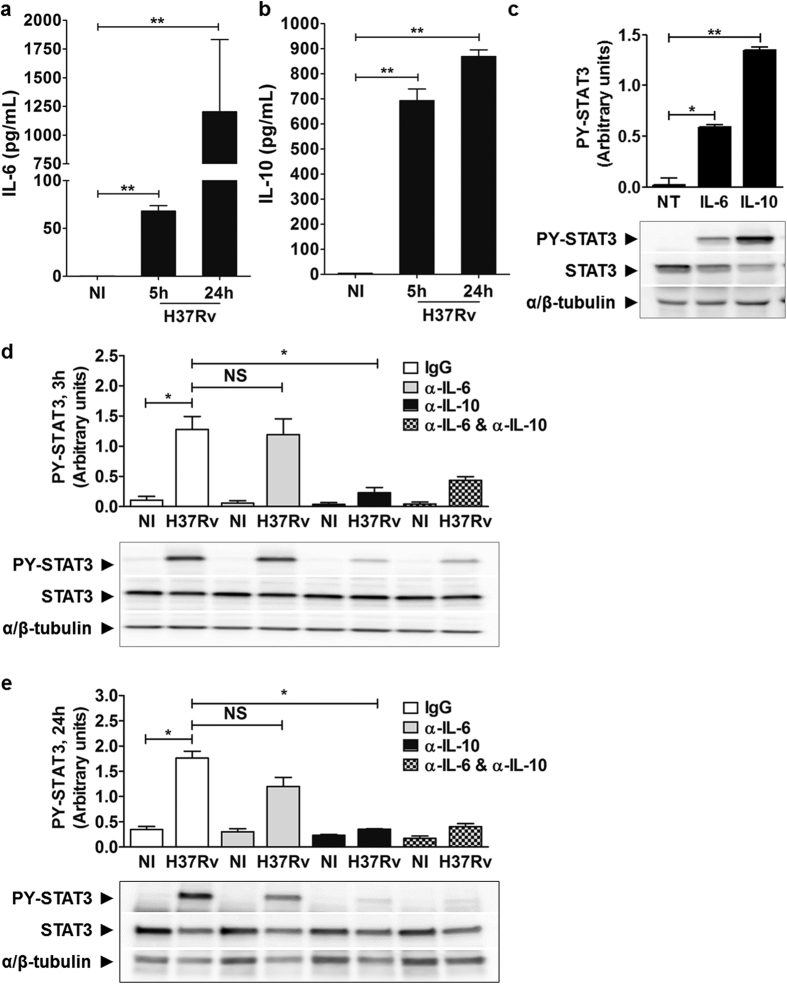
Activation of STAT3 mainly occurs through IL-10 signaling. (**a**) IL-6 and (**b**) IL-10 release was quantified from supernatants collected from uninfected or H37Rv-GFP-infected hMΦ at MOI 2 for 5 h or 24 h, using Cytokine Human 30-Plex array (Life Technologies). Reported values represent the average concentrations of cytokine released ± SEM from two independent donors, each tested in duplicate. (**c**) hMΦ were treated with 20 ng/mL IL-6 or 10 ng/mL IL-10 for 30 min. Non-treated cells (NT) were used as a negative control. STAT3 activation in hMΦ was analyzed by immunoblotting using anti-PY^705^-STAT3 antibody. (**d,e**) hMΦ were infected with H37Rv-GFP at a MOI of 2 (H37Rv) for 3 h (**d**) or 24 h (**e**) in the presence of 10 μg/mL of control antibody (IgG Ctrl), neutralizing anti-IL-6 (α-IL-6), neutralizing anti-IL-10 (α-IL-10) or both (α-IL-6 + α-IL-10). For each condition, uninfected cells (NI) were used as a negative control. Total protein extract were probed with anti-STAT3 and anti-α/β-tubulin were used to confirm gel loading. Reported values represent the means of 3 independent experiments ± SEM and correspond to the relative STAT3 phosphorylation. Full-length blots are presented in Supplementary Fig. S12. Asterisks indicate the statistically significant differences between compared conditions, calculated using the Student t-test (*p < 0.05, **p < 0.01, NS: no-significant).

Originally, STAT3 was described as a transcription factor activated *via* cytokine receptors sharing the common signal-transducing subunit gp130, such as the IL-6 receptor, but also *via* stimulation of cytokine receptors harboring common STAT3 docking motif (YxxQ), like the IL-10 receptor^20^,^21^,^22^,^23^,^24^. We found that IL-6 and IL-10 were both released by hMΦ at 5 hour post-infection by *M. tuberculosis* and remained significantly detectable in the supernatant 24 hours post-infection (Fig. 2a,b, respectively). We then showed that stimulation of hMΦ with both purified human IL-6 and IL-10 led to a significant activation of STAT3 phosphorylation (Fig. 2c). To identify which of the two interleukines specifically stimulates STAT3 activation during *M. tuberculosis* infection, hMΦ were treated with blocking anti-IL-6 and/or anti-IL-10 antibodies before being infected with H37Rv (Fig. 2d,e). STAT3 activation remained largely unaffected in hMΦ treated with neutralizing anti-IL-6 (Fig. 2d,e, Supplementary Fig. S4b). We verified that neutralization by anti-IL-6 antibody was effective in our samples, by testing its ability to block STAT3 activation upon exogenous addition of pure IL-6. Addition of neutralizing anti-IL-6 strongly resulted in the inhibition of STAT3 phosphorylation induced by IL-6 (Supplementary Fig. S4a), ruling out a possible experimental error. Importantly, neutralization of IL-10 markedly blocked STAT3 activation suggesting that early STAT3 signaling is mainly driven by IL-10 (Fig. 2d,e, Supplementary Fig. S4b). Corroborating this, we ascertained that *M. tuberculosis* was unable to induce STAT3 phosphorylation in bone-marrow derived macrophages (BMDM) from IL-10 KO mice in contrast to BMDM from WT mice (Supplementary Fig. S4B,C). These results demonstrate that secretion of IL-10 following *M. tuberculosis* uptake is essential for STAT3 activation in human macrophages.

### STAT3 represses the transcription of inflammatory cytokines upon infection of macrophages by *M. tuberculosis*

STAT3 has been described as a strong modulator of cellular pathways^25^. To analyze the impact of STAT3 in hMΦ during the first hours after *M. tuberculosis* infection, the expression of 84 genes involved in cellular pathways, including apoptosis, necrosis/necroptosis and autophagy, was investigated in hMΦ silenced for STAT3 by using RT-qPCR (Supplementary Table S2). We first ascertained that STAT3 silencing did not impact the bacterial uptake in our settings. The number of *M. tuberculosis* Colony Forming Units (CFUs) that was taken up 4 hours after infection was similar for hMΦ silenced for STAT3 (siSTAT3) and for hMΦ transfected with control siRNA (scramble) (Supplementary Fig. S5a). To avoid any bias or misinterpretation due to hMΦ stress following *M. tuberculosis* uptake, we analyzed the gene expression 24 hours post-infection, when STAT3 is still activated (Fig. 1a). Scramble- and siSTAT3-hMΦ were then infected with H37Rv at a MOI of 1, and mRNA were extracted at 24 hours^26^. In these conditions, expression of STAT3 and PY-STAT3 upon infection was fully abrogated (Supplementary Fig. S5b–d). For each gene tested, mRNA levels from scramble and siSTAT3 hMΦ after infection were normalized to that of non-infected scramble-hMΦ (Supplementary Fig. S6, Supplementary Table S2). Six genes were found modulated by STAT3 in absence of infection and were then excluded from the analysis. Upon *M. tuberculosis* infection, 43 genes had their expression level modified in both siSTAT3 and scramble groups relative to scramble group in absence of infection and 16 genes were finally selected based on a p-value < 0.05 (Supplementary Fig. S6a,b and Table S2). Finally, 9 genes were specifically modulated by STAT3 upon infection. For instance, the anti-apoptotic genes *TNFRSF11B*, also known as Osteoprotegerin (*OPG*), and Baculoviral IAP repeat containing 3 (*BIRC3*) were less expressed in siSTAT3 compared to scramble (Supplementary Fig. S6c). For the pro-necroptosis deubiquitinase *CYLD*^27^, its expression was up-regulated upon STAT3 silencing (Supplementary Fig. S6c). Importantly, the mRNA levels of TNF-α, and more surprisingly, IFN-γ were strongly increased upon STAT3 silencing (5.5 and 7.5 fold, respectively) (Supplementary Fig. S6b,c). The up-regulation of both TNF-α and IFN-γ mRNA suggests that STAT3 represses inflammatory cytokine production at the transcriptional level, limiting acute inflammation at the site of infection.

### STAT3 prevents early inflammatory response during *M. tuberculosis* infection

**Figure 3:**
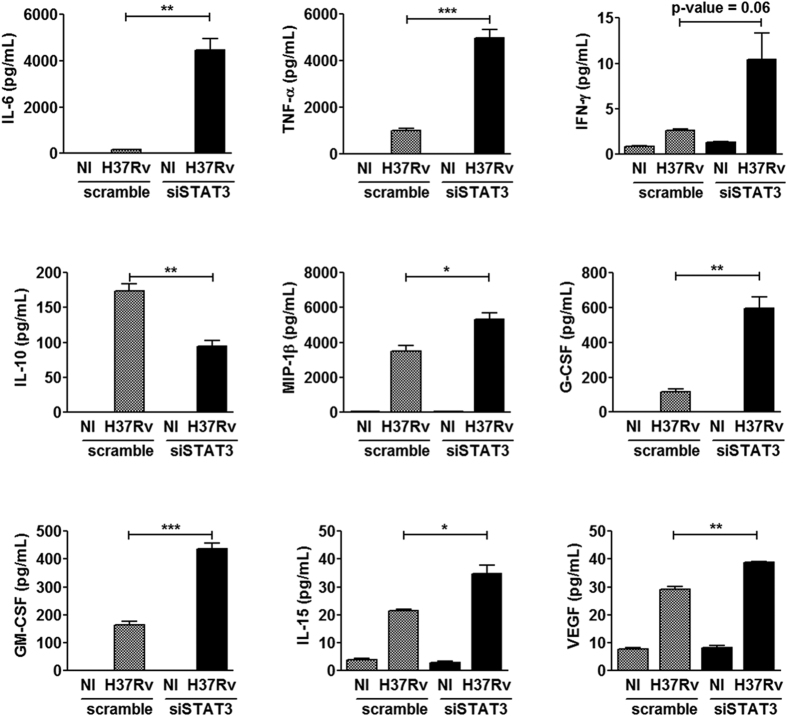
STAT3 prevents early inflammatory response during *M. tuberculosis* infection. Quantification of the differential release of 30 cytokines, measured 24 hours post-infection, in siSTAT3 and scramble hMΦ. In the two conditions, non-infected cells (NI) were used as negative controls. Values represent average concentrations of the indicated cytokines released ± SEM. Average concentrations were representative of 2 independent experiments using pooled macrophages from two donors, each tested in triplicate. Asterisks indicate the statistically significant differences between supernatants collected from siSTAT3 and scramble hMΦ, calculated using the Student t-test (*p < 0.05, **p < 0.01, ***p < 0.001).

To gain more insight in the STAT3-controlled cytokines, we quantified the release of 30 different cytokines by scramble- and siSTAT3-hMΦ, 24 hours after infection with *M. tuberculosis* H37Rv (Supplementary Table S3). After infection, a total of 9 cytokines displayed a significant difference between scramble and siSTAT3 samples, while retaining the same profile between uninfected cells transfected with scramble and siSTAT3 (Fig. 3, Supplementary Table S2). Inhibition of STAT3 signaling during infection led to an increase of the inflammatory cytokine secretion, IL-6 (26 fold) and TNF-α (5 fold), compared to control infected macrophages (Fig. 3). To a lesser extent, the level of secreted IFN-γ was found to be weakly increased (4 fold, p-value < 0.06) in infected-hMΦ siSTAT3 compared to infected-hMΦ scramble. Consistently with the strong increase of inflammatory cytokines, we found that the release of IL-10 was decreased by two-fold in the siSTAT3 group compared to the scramble group (Fig. 3).

STAT3 silencing also led to an increase in cytokines involved in the recruitment, proliferation and/or differentiation of hematopoietic and lymphoid cells, such as MIP-1β (1.5 fold), G-CSF (5 fold), GM-CSF (2.5 fold) and IL-15 (1.6 fold) (Fig. 3). These factors may help the recruitment of specialized immune cells, such as dendritic cells, neutrophils and lymphocytes, thereby impacting on the outcome of the innate as well as the adaptive immune responses to the bacilli. Finally, the amount of released VEGF was significantly higher in the supernatants from the siSTAT3 group compared to the control group (Fig. 3). This increase in VEGF secretion could be the consequence of a more pronounced inflammatory response observed in the siSTAT3 group. VEGF secretion has also been related to macrophage activation^28^,^29^. Altogether, our results demonstrate that STAT3 is a dominant modulator of the immune response, controlling inflammation in *M. tuberculosis*-infected macrophages, which may be beneficial for the establishment of an intracellular niche for *M. tuberculosis* survival at the site of infection.

### STAT3 signaling prevents nitric oxide production in both *M. tuberculosis*-infected and NI-BC macrophages

**Figure 4:**
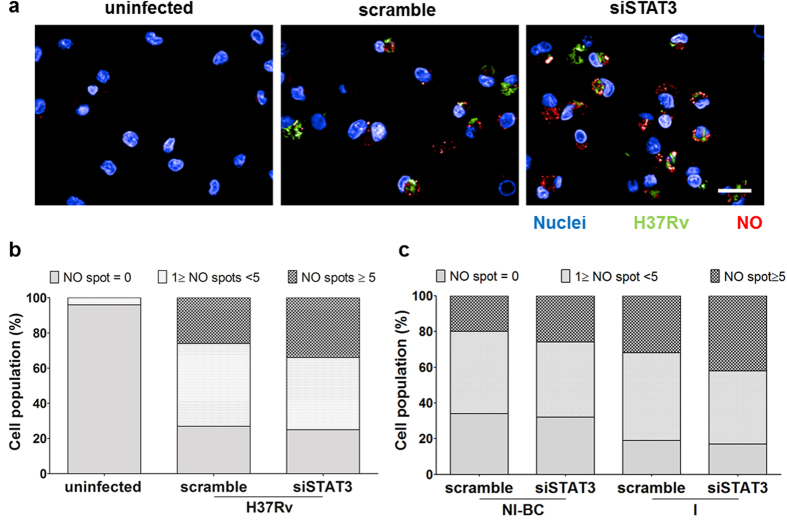
STAT3 signaling prevents nitric oxide production in both *M. tuberculosis*-infected and bystander uninfected macrophages. Quantification by fluorescence of intracellular NO production, measured 24 hours post-infection, in siSTAT3 and scramble hMΦ. In the two conditions, non-infected cells (NI) were used as negative controls. (**a**) Typical images taken using automated confocal microscope with 63X water lens. The scale bar represent 20 μm. Macrophage nuclei were stained using DAPI (blue), H37Rv-GFP are visualized in green and NO in red (I: Infected cells; NI-BC: Non-Infected Bystander Cells). (**b**,**c**) Distribution of cell population based on NO spots detection. Data represent the average of triplicate values from two different donors.

Production of free radical nitric oxide (NO) is one anti-bacterial strategy deployed by macrophages to limit the proliferation of intracellular pathogens such as *M. tuberculosis*. It was previously shown that, *M. tuberculosis* regulates the amount of NO through a mechanism involving STAT proteins^19^. In this context, we hypothesized that, in human macrophages, the early activation of STAT3 signaling prevents an acute production of NO. To study the NO production, hMΦ were infected with GFP-expressing *M. tuberculosis* H37Rv (H37Rv-GFP) at a MOI of 1 for 24 hours. On one hand, the concentration of nitrite in cell supernatant was monitored using the Griess reagent. On the other hand, the expression of inducible nitric oxide synthase (NOS2) was quantified by RT-PCR (Supplementary Fig. S7a,b). For the two different hMΦ donors tested, an increased concentration of Nitrites in siSTAT3-hMΦ supernatants was found relative to scramble-hMΦ as well as a higher expression of NOS2 mRNA. To confirm our findings, we then measured the production of NO by fluorescence microscopy using an NO specific dye (Fig. 4a,b)^30^. Image-based analysis allowed us to quantify the NO area per cells (Supplementary Fig. S2, Table S1). First, by studying the whole cell population, we found that *M. tuberculosis*-infected siSTAT3-hMΦ had a higher production of NO compared to scramble-hMφ. As control, in absence of bacteria, there is no NO produced by uninfected hMΦ (Fig. 4a,b, Supplementary Fig. S8a,b). We showed above that the IL-10-dependent activation of STAT3 occurred in hMΦ containing intracellular bacilli as well as in non-infected bystander macrophages (NI-BC, Fig. 1). Similarly, we found a significant increase of NO expression in siSTAT3-NI-BC compared to scramble-NI-BC (Fig. 4c, Supplementary Fig. S8c,d). The involvement of STAT3 in the regulation of NO production, in both infected and non-infected bystander macrophages further support the key role of STAT3 in the control of host defenses during *M. tuberculosis* infection. This led us to determine the effect of STAT3 on the tubercle bacilli intracellular replication.

### STAT3 signaling is required for the intracellular survival of *M. tuberculosis* within macrophages

**Figure 5:**
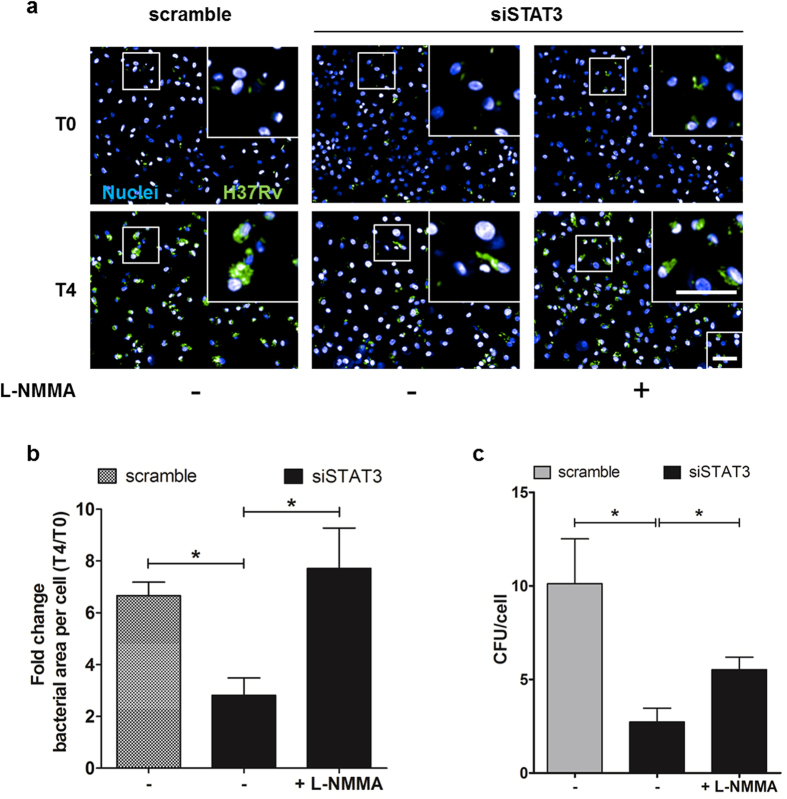
STAT3 signaling is required for the intracellular replication of *M. tuberculosis* within macrophages. (**a,b**) siSTAT3 and scramble hMΦ were infected with H37Rv-GFP at a MOI of 0.5 for 4 days in presence (+) or in absence of 1 mM of iNOS inhibitor L-NMMA. (**a**) Confocal images of H37Rv-infected siSTAT3 and scramble hMΦ. Images were taken just after uptake (T0) and 4 days post infection. (T4). Nuclei are labeled with DAPI (Blue) and GFP-bacteria are visualized in green. Scale bar represent 50 μm. (**b**) Image-based quantification of intracellular growth of *M. tuberculosis* in scramble-hMΦ or siSTAT3-hMΦ treated (+) or not (−) with iNOS inhibitor L-NMMA. Values represent the growth *M. tuberculosis* expressed by the ratio T4/T0 of the bacterial area per cell. Average values ± SD are representative of two independent experiments. Asterisks indicate the statistically significant differences between compared conditions, calculated using the Student t-test (*p < 0.05). (**c**) siSTAT3- or scramble-hMΦ were infected with H37Rv-GFP at a MOI of 0.5 for 4 days in absence (−) or in presence 1 mM L-NMMA. Cells were then lysed and the titer of intracellular H37Rv-GFP was determined by CFU counting and normalized to the number of cells for each condition tested (CFU/cell). Average values ± SD are representative of two independent experiments performed in triplicates. Asterisks indicate the statistically significant differences between compared conditions, calculated using the Student t-test (*p < 0.05).

The role of STAT3 in the control of inflammatory signaling and NO production suggests that upon *M. tuberculosis* infection STAT3 signaling, most likely benefits to the pathogen. To test whether the inhibition of STAT3 activation may affect the intracellular proliferation of *M. tuberculosis*, hMΦ transfected with siSTAT3 and infected with H37Rv-GFP. Just after bacterial uptake (T0) and four days post infection. (T4), macrophages nuclei were labelled with DAPI and images were acquired on confocal microscope. For each condition, the intracellular *M. tuberculosis* growth was determined by the ratio of the bacteria area per cell at T4 relative to T0 using our established image based analysis (Supplementary Figs S2 and S10, Table S1)^31^,^32^. SiSTAT3-hMΦ had less bacterial load compared to scramble control (Fig. 5a,b). This correlated with a decrease in the number of CFU per cell in siSTAT3-hMΦ compared to scramble ones (Fig. 5c). Furthermore, addition of nitric oxide synthase (NOS) inhibitor L-NMMA (+) on siSTAT3 samples restored bacterial replication thus showing that the impact of STAT3 signaling on bacterial replication is mediated by the repression of NO synthesis (Fig. 5a,b,c). Taken together, our results support that, in *M. tuberculosis*-infected macrophage, STAT3 signaling promotes intracellular bacterial replication.

## Discussion

The ability of *M. tuberculosis* to precociously manipulate macrophage defense mechanisms is a decisive event for the outcome of TB infection, but the orchestration of the pathways involved in this process is still unclear.

STAT family protein appears to be key host molecules playing a crucial role in the achievement of *M. tuberculosis* immunity. In particular, STAT3 is now considered as one major controller of the outcome of infection with *M. tuberculosis*^19^,^33^. STAT3 has been previously described to play multiple roles during *M. tuberculosis* infection. Indeed, STAT3 is not only involved in both pro- and anti-inflammatory response in myeloid cell but also in the differentiation and proliferation of inflammatory T-cells. However, the mechanism by which STAT3 modulates macrophage response, at the early stage of the infection, is still incomplete. We demonstrate here that the early activation and nuclear translocation of STAT3 occur in primary human macrophages in response to *M. tuberculosis* H37Rv infection and that STAT3 signaling spreads through cell-to-cell communication. Although IL-6 and IL-10 were both secreted in the first hours of infection, IL-10 mainly mediates the activation of STAT3 signaling in infected macrophages. It was previously reported that the distinction between IL-6- and IL-10-dependent STAT3 signaling was differentially modulated by SOCS3 which preferentially inhibit IL-6 receptor signaling^23^,^34^,^35^. In contrast to IL-10 receptor (IL-10R), IL-6 receptor (IL-6R) carries the signal transducer gp130 involved in the STAT3 phosphorylation. IL-6 signaling is selectively inhibited owing to the binding of SOCS3 to the IL-6R subunit gp130, which consequently blocks the IL-6-dependent activation of STAT3 while IL-10-dependent activation of STAT3 is not restricted by SOCS3^36^. Similarly as STAT3, SOCS3 is expressed in *M. tuberculosis*-infected human macrophages as early as 3 hours post-infection and remains highly detectable up to 24 hours post-infection (Supplementary Fig. S9). The IL-10-dependent STAT3 activation following *M. tuberculosis* uptake suggests that the initial wave of STAT3 activation from IL-6 may be quickly blocked by SOCS3, giving priority to STAT3-induced anti-inflammatory response through IL-10 signaling. In the mouse model, the IL-10-dependent anti-inflammatory response requires STAT3 to activate the expression of genes involved in the expression of inflammatory genes^37^. Similarly to the mouse model, we demonstrated in human macrophages the crucial role of IL-10 in early STAT3 signaling, which then mediates an anti-inflammatory response aiming to antagonize pro-inflammatory signals^34^,^37^. To avoid any misinterpretation due to the macrophage stress induced by *M. tuberculosis* uptake, we observed the consequence of STAT3 silencing 24 hours post-infection. Accordingly, we found that abolition of early STAT3 signaling in macrophages strongly modifies the gene expression and the immune responses. In particular, abrogation of STAT3 signaling during *M. tuberculosis* infection led to an enormous increase in TNF-α mRNA levels, correlating with a higher secretion. Surprisingly, STAT3 silencing also led to a strong increase of IFN-γ mRNA production which contrasted with a really low detection of secreted IFN-γ. The ability of myeloid cells, including primary macrophages, to produce IFN-γ is controversial. One of the major concerns regarding the detection of IFN-γ in primary macrophages is the possible contamination of macrophage cultures by lymphocytes. However, in our study, the method used to purify human macrophages provides a CD14+/CD206+ macrophage purity >99%. Moreover, several studies including some performed at single-cell level; point out the ability of macrophages and dendritic cells to produce IFN-γ in response to various stimuli^38^,^39^.

In the early stage of infection, STAT3 also strongly represses the production of inflammatory cytokines such as IL-6, while enhancing secretion of anti-inflammatory IL-10. STAT3 was previously reported to be recruited by the IL-10 promoter and to activate its transcription^40^. Furthermore, the production of inflammatory cytokines, in response to LPS, has been shown to be dramatically increased in STAT3-deficient murine macrophages while the suppressive effect of IL-10 on cytokine production was abolished^41^. These latter points supported the crucial role of IL-10/STAT3-mediated anti-inflammatory response in *M. tuberculosis*-infected human macrophages. We also found that STAT3 controls the secretion of multiple immune factors such as MIP1β, IL-15, G-CSF and GM-CSF, which are involved in recruitment, proliferation and differentiation of dendritic cells, neutrophils and lymphocytes. In particular, G-CSF controls the production and mobilization of neutrophils. In addition to macrophages, neutrophils also constitute a first line of defense against *M. tuberculosis* in lung^19^. The STAT3 –dependent repression of G-CSF release by macrophages suggests that STAT3 limits early migration of neutrophils at the infection site supporting that STAT3 may be essential for the coordination of innate and adaptive immune responses.

The IL-10 dependent anti-inflammatory response was proposed to be linked with the ability of *M. tuberculosis* to evade the immune response and mediate long-term infection in the lungs^42^. Virulent *M. tuberculosis* produced several factors suppressing various cell death pathways such as apoptosis, pyroptosis or autophagy^42^,^43^,^44^. On the macrophage side, inflammatory cytokines, in particular TNF-α, have been shown to increase macrophage defense against intracellular *M. tuberculosis* by inducing apoptosis^45^. We thus investigated the role of STAT3 in the regulation of genes involved in cell death/survival pathways. At the early stage of *M. tuberculosis* infection, STAT3 signaling modulates macrophage response at transcriptional level and may participate to the control of macrophage stability. In particular, we showed that STAT3 negatively regulated expression of pro-necroptotic deubiquitinase Cylindromatosis (*CYLD*) and the pro-apoptotic Bcl2 modifying factor (*BMF*) in infected macrophages. In contrast, STAT3 also induced an up-regulation of anti-apoptotic genes such as Baculoviral IAP repeat containing 3 (*BIRC3*) and *TNFRSF11B*, also called Osteoprotegerin (*OPG*). Surprisingly, we did not observe a clear increase of macrophage death in absence of STAT3 signaling suggesting that other components are responsible for the complex regulation of infected-macrophage longevity. However, the implication of these genes upon *M. tuberculosis* macrophage infection has not been previously reported, and their potential involvement in the regulation of human macrophage longevity during *M. tuberculosis* infection has to be further studied. Nonetheless, STAT3 has previously been described, in mouse model, as a strong modulator of cell death pathways, acting either as pro- or anti-apoptotic inducer suggesting that its pleiotropic function is conserved in human macrophages^25^.

Among the anti-mycobacterial molecules produced by host macrophage, free radical NO is one of the most efficient ones capable to restrain *M. tuberculosis* infection. In mice, NO plays a key role in innate immunity against *M. tuberculosis*^46^. Induction of NO production in IFN-γ- or L-arginine-activated macrophages led to *M. tuberculosis* growth inhibition and killing^47^. Consistently, *M. tuberculosis*-infected mice treated with nitric oxide synthase inhibitors presented higher bacterial burden, mortality and tissue damages^48^. In addition, *M. tuberculosis*-infected iNOS KO mice presented a higher risk of dissemination and mortality compared to control mice^49^,^50^. In contrast to murine model, the role of NO in the inhibition of *M. tuberculosis* growth is controversial in human^51^. For instance, neither exogenous IFN-γ treatment nor competitive inhibition of nitric oxide synthase, by L-NMMA, have any effect on mycobacterial growth in human primary macrophages^52^,^53^. However, a growing body of evidence suggests that NO production by *M. tuberculosis*-infected human macrophages or macrophage-like cell lines induces bacteriostatic activity against *M. tuberculosis*^54^,^55^,^56^. NO production in infected-alveolar macrophages from healthy control subjects was also shown to be correlated with inhibition of *M. tuberculosis* intracellular growth^57^. However, it is important to note that the authors observed marked variation between healthy subjects suggesting genetic background may influence antimycobacterial activity of NO. Although INF-γ-induced NO in human macrophage provides a questionable effect on the *M. tuberculosis* intracellular growth, the induction of NO by exogenous vitamin D, or in combination with INF-γ, presented a potent antimycobacterial activity^55^. This suggests that *M. tuberculosis* susceptibility to NO may be dependent on the molecular cascade engaged. For instance, the antimycobacterial activity of Dipterinyl calcium pentahydrate (DCP), have been correlated with a MIP1β-dependent production of NO in human macrophages^56^.

We demonstrated here that STAT3 repressed the expression and secretion of both IFN-γ and MIP1β. Accordingly, we showed that STAT3 limited the production of intracellular NO in both *M. tuberculosis*-infected and non-infected bystander human primary macrophages. Corroborating this, knock-down of STAT3 resulted in a significant increase of intracellular NO production which is correlated with a significant decrease of intracellular mycobacterial load.

According to our data, we propose that, in *M. tuberculosis*-infected human macrophages, STAT3 regulates the expression of multiple cellular pathways, limits macrophage inflammatory response and controls NO production. The pleiotropic functions driven by STAT3 tend toward curbing macrophage response, which favors *M. tuberculosis* intracellular adaptation. Furthermore, in absence of STAT3 signaling, virulent *M. tuberculosis* appeared to be more sensitive to NO suggesting that, in human macrophages, STAT3 may modulate *M. tuberculosis* susceptibility to nitric oxide.

## Materials and Methods

### Antibodies and reagents

Polyclonal rabbit anti-PY^705^-STAT3 (#9145), anti-STAT3 (#4904) and anti-α/β-tubulin (#2148) were purchased from Cell Signaling Technology. For western blotting, the antibodies were probed using secondary donkey anti-rabbit antibodies, conjugated with horseradish peroxidase (Jackson Immunoresearch, #711-035-152). For immunofluorescence, the antibodies were probed using secondary donkey anti-rabbit antibodies conjugated to Rhodamine Red X (Jackson Immunoresearch, #711-295-152). Cell nuclei were fluorescently labelled using DAPI (Sigma-Aldrich, #D9564) or Syto 60 (Life Technologies, #S11342). Purified human IL-10 (#130-093-947), IL-6 (#130-095-365) and pure grade neutralizing anti-IL-6 (#130-096-093) and anti-IL-10 (#130-096-041) antibodies were purchased from Miltenyi Biotech. Nitric oxide synthase inhibitor L-NMMA (#0771, Tocris) was suspended in desionized water at the concentration of 50 mM.

### Mammalian cells

Murine Raw 264.7 cells (ATCC TIB-71) were grown to 60–80% confluence in RPMI 1640 medium (Difco) supplemented with 10% heat-inactivated FBS (Life Technologies). Human CD14^+^ monocytes were purified from whole blood using CD14 microbeads (from Myltenyi Biotech) as previously described^32^. CD14^+^ monocytes were then differentiated into macrophages by 6-days incubation in RPMI 1640 complemented with 10% FBS and 40 ng/mL human Macrophage Colony Stimulating Factor (hM-CSF) (Miltenyi Biotech) at 37 °C in 5% CO2. 6 days after hM-CSF differentiation, CD14 macrophages (hMφ) were labeled with anti-CD14- AlexaFluor488 and anti-CD206-APC (#562689 and #550889, BD) and analyzed by Flow cytometry. CD14-AlexaFluor488-positive cells (CD14+) were detected using laser: 488 nm/filter: 500–550 and CD206-APC-positive cells (CD206+) were detected using laser: 561 nm/filter 655–730. Analysis revealed a purity >99% of CD14+/CD206+ hMφ. Balb/c (WT), IL-10 KO (Balb/c background) mice were differentiated for 6 days in RPMI 1640 complemented with 10% FBS and with 10% of culture supernatant from L929 overexpressing murine M-CSF. (Ethics statement from CEEA Nord-Pas de Calais N° 00579.01). Cells were then plated one day prior to infection.

### Mycobacterial strains and cell infection

A recombinant strain of *M. tuberculosis* H37Rv expressing the enhanced green fluorescent protein (H37Rv-GFP) was obtained by transformation of an integrative plasmid kindly provided by Dr. Nathalie Winter^58^ (Unité de Génétique Mycobactérienne, Institut Pasteur, Paris). In this construct, the *Aequoria victoria egfp* gene was inserted into an Ms6-derived integrative vector downstream of the mycobacterial promoter pBlaF. An EGFP-positive transformant was selected on hygromycin and grown in Middlebrook 7H9 medium (Difco) supplemented with 10% oleic acid-albumin-dextrose-catalase (OADC, Difco), 0.2% glycerol, 0.05% Tween 80, and 50 μg/ml hygromycin (all from Sigma-Aldrich, St. Louis, MO, USA) until the exponential phase was reached. Bacilli were washed with Dulbecco’s Phosphate Buffered Saline (DPBS free from MgCl_2_ and CaCl_2_, Gibco) resuspended in 10 mL of RPMI 1640 medium containing 10% FBS and decanted for 1 h at room temperature to allow bacterial aggregates to sediment. Bacterial titer was determined by measuring the GFP fluorescence on a Victor Multilabel Counter (Perkin Elmer). The concentration of the suspension was calculated using a reference regression line displaying RFU value = f (CFU value) that had been generated prior to the experiment on another culture that had been prepared in the same conditions. The bacterial suspension was diluted at the required titer in RPMI 1640 supplemented with 10% FBS prior to infection. Just after infection, the level of intracellular bacteria was then ascertained by automated confocal microscopy and dedicated image analysis of the field area whose GFP intensity is above a certain threshold. Alternatively, CFU were determined to ascertain the bacterial uptake.

### siRNA Transfection

siRNA transfection in primary hMΦ was adapted from the protocol previously described^26^. Briefly, 5 days after differentiation with human M-CSF (40 ng/mL), primary CD14+ hMΦ were collected for reverse transfection using the HiPerFect transfection reagent from Qiagen. For STAT3 silencing, 50 nM of pooled STAT3 siRNA from Dharmacon were used (D-003544-02, D-003544-03, D-003544-04, D-003544-19). As control, macrophages were transfected with 50 nM of pooled Non-targeting siRNA (scramble). Transfected cells were then incubated for 3 days in RPMI 1640 supplemented with 10% FBS and hM-CSF 20 ng/mL at 37 °C in 5% CO_2_. Transfected cells were then infected or not with *M. tuberculosis,* and samples were collected at the indicated time points.

### CFU determination

Cells were washed before lysis with DPBS-0.1% Triton X-100 buffer. Serial dilutions were performed in DPBS and plated onto 7H11 agar plates supplemented with 10% OADC. CFUs were then calculated after a three week growth.

### Supernatant transfer assay

hMΦ were first infected with *M. tuberculosis* H37Rv at a MOI of 2 for 3 hours, then the supernatant was collected and filtered using a 0.22 μM PVDF filter to remove any residual bacteria. Non-infected hMΦ were further incubated with filtered supernatant for 1.5 hour. Cells were collected, and STAT3 activation was determined by western blotting.

### Electrophoresis and immunoblotting

Cells were washed with DPBS (Difco), lysed using 62.5 mM Tris-HCl pH 6.8, 10% glycerol, 4% SDS, 0.025% bromophenol blue and 1.5% β-mercaptoethanol, and boiled at 100 °C for 15 min. The proteins were resolved by SDS-PAGE in precast acrylamide gels 4–15% (BioRad) and electrotransferred onto a polyvinylidene difluoride (PVDF) membrane. The PVDF membranes were blocked in DPBS, 0.1% Tween 20, 5% BSA and probed overnight with the appropriate antibodies. Blots were then incubated with horseradish peroxidase-conjugated secondary antibodies (Jackson Immunoresearch), followed by detection using chemiluminescence, according to the manufacturer’s instructions (Immobilon, Millipore). The chemiluminescent signal was detected using the LAS 3000 Pro Bio Imaging Analyser. Spot fluorescence intensities were quantified using ImageJ software.

### Neutralization of IL-6 and IL-10 activity

Primary CD14^+^ hMΦ were infected for 3 hours with *M. tuberculosis* H37Rv at a MOI of 2, in the presence of 10 μg/mL control IgG, neutralizing anti-IL-6 (α-IL-6) or anti-IL-10 (α-IL-10) antibodies. Phosphorylation of STAT3 was analyzed by electrophoresis and immunoblotting as described above.

### Immunofluorescence

After infection, hMΦ and human *in vitro* granuloma were fixed with 10% neutral buffered Formalin solution (Sigma-Aldrich) for 30 min and permeabilized with cold methanol for 10 min at −20 °C. Cells were then incubated with blocking buffer (DPBS, 1% FBS) for 30 min prior to overnight incubation at 4 °C with rabbit anti-PY^705^-STAT3. Cells were then washed 3 times with DPBS and incubated with anti-rabbit-RRX for 1 hour at room temperature. Cells were again washed 3 times with DPBS and incubated for 10 min with 2.5 μg/mL DAPI in DPBS. The buffer was replaced by D-PBS containing 1% FBS. Plates were sealed and stored at 4 °C.

### Intracellular growth assay

hMΦ were transfected with scramble control siRNA or STAT3 siRNA. Cells were then infected with fluorescent *M. tuberculosis,* in presence of vehicle H_2_O or NOS inhibitor L-NMMA at the final concentration of 1 mM and incubated at 37 °C in atmosphere containing 5% CO_2_ for 2 hours. Cells were then washed and treated with Amikacin 50 μg/mL to remove residual extracellular bacteria. Cells were finally washed with PBS and incubated in fresh complete culture medium containing vehicule H_2_O or 1 mM L-NMMA. Just after bacterial uptake (T0) and after 4 days post-infection (T4), cell were fixed with 10% neutral buffered Formalin solution for 30 min. Nuclei were stained using DAPI and pictures were recorded by automated confocal fluorescence microscopy. The numbers of cells and the bacterial areas in the well were determined by image-based quantification^32^. Intracellular bacterial growth was then quantified by the ratio Bacterial area per cell T4/T0 (see “Image-based analysis” section).

### Intracellular nitric oxide (NO) quantification

siSTAT3 and scramble hMΦ were infected with H37Rv-GFP at a MOI of 1 for 24 h. Intracellular NO was stained with NO specific probe (O-phenylenediamine) from Enzo (ENZ-51013-200). In presence of NO, O-phenylenediamine oxidizes to the corresponding aryl triazole providing a robust switch for NO detection under aerated conditions, as the fluorescent triazole product is not formed by reaction with superoxide, hydrogen peroxide, or peroxynitrite^30^. hMΦ were incubated for 2 hours at 37 °C with RPMI 1640 medium containing 10% FBS, NO probe (dilution 1:400) and Hoechst 0.5 μg/mL. Cells were then washed twice with 1X washing buffer provided by the manufacturer. For image acquisition, RPMI 1640 medium without red phenol was used. Images were then analyzed using the software Columbus from Perkin Elmer Company. Nuclei, cytoplasm and NO spots inside the cells were sequentially segmented as explained in “Image analysis” section and illustrated in Supplementary Fig. S2. Per cell data have been generated to quantify the number of NO spots per cell. Boxplots were plotted for each condition and Mann-Whitney tests performed using the R software. Concentration of Nitrites in cell supernatant from siSTAT3 and scramble-hMΦ was determined using the Greiss reagent (Molecular Probes #G-7921) following the manufacturer’s protocol. A reference curve was established using Nitrite standard (range: 1 μM to 50 μM).

### Image acquisition

Images were acquired using an automated fluorescent confocal microscope (OPERA, PerkinElmer) equipped with a 20X (NA 0.70) or 63X (NA 1.2) water lens. The confocal microscope was equipped with 405, 488, 561 and 640 nm excitation lasers. The emitted fluorescence was captured using 3 cameras associated with a set of filters covering a detection wavelength ranging from 450 to 690 nm.

### Image-based analysis

After acquisition, images from the automated confocal microscope were analyzed using a dedicated script developed using the image-analysis software Acapella 2.6 (PerkinElmer). (Supplementary Fig. S2, Table S1).

#### Cell detection and M. tuberculosis intracellular growth

Nuclei and cytoplasm were both detected by a local intensity detection algorithm applied on the DAPI channel (nuclei: maximal local intensity; cytoplasm: minimal local intensity). A spot detection algorithm based on the GFP channel was applied for the detection of intracellular *M. tuberculosis*-GFP. A manual threshold method, using non-infected wells, was applied to determine the background threshold. These GFP spots were defined as region of interest (ROI) for the measurement of bacterial intensity and area in pixels. In each well; two sub-populations were determined: (i) cell containing bacteria (infected cells) and (ii) cell without bacteria (non-infected bystander cells (NI-BC)). The intracellular bacterial growth was quantified by the ratio of intracellular bacterial area (pixel) per cell between T0 (after uptake) and 4days post-infection (T4). Data were then presented as Fold change on bacterial area/cell (T4/T0).

#### STAT3 nuclear translocation

Cell sub-populations (infected cells and NI-BC cells) were detected as described above. For STAT3 nuclear translocation, cell nuclei were defined as ROI for the calculation of intensity properties in PY-STAT3 channel. A manual threshold method, using non-infected wells, was applied to determine the background threshold. For each nucleus, intensity properties (Mean, Max, Sum) were measured to detect cells with PY-STAT3 nuclear translocation. Cells were then split in 4 sub-populations: (i) infected cells with PY-STAT3 nuclear translocation, (ii) infected cells without PY-STAT3 nuclear translocation (iii) NI-BC with PY-STAT3 nuclear translocation and (iv) NI-BC without PY-STAT3 nuclear translocation.

#### Intracellular nitric oxides (NO) detection

Cell sub-populations (infected cells and NI-BC cells) were detected as described above. For the NO detection, cytoplasm was defined as ROI. We applied an algorithm that detects round shaped spots with a given maximum radius on the NO channel. A manual threshold method, using non-infected wells, was then applied to determine the basal level of NO. For each NO spot detected intensity properties (Mean, Max, Sum) and NO area in pixel were calculated. These parameters were measured for all cells (single cell analysis) and data were then plotted (box plot) and expressed as, (i) number of NO spots in infected cells and NI-BC, or (ii) NO area/infected cell and NO area/NI-BC.

### Human 30-Plex cytokine assay

Primary hMΦ were infected with *M. tuberculosis* H37Rv at a MOI of 1. Five or 24 hours after *M. tuberculosis* infection, cell culture supernatants were filtered using a 0.22 μM PVDF filter, sampled and stored at −80 °C until analysis. Cytokine concentrations were quantified using Human cytokine Magnetic 30-plex kit (Life Technologies) accordingly to the manufacturer’s protocol.

### mRNA purification and data analysis from RT^2^ Profiler PCR Array

After infection, hMφ were washed once with DPBS and mRNA was purified by using the RNeasy purification kit from QIAGEN, according to the manufacturer’s recommendations. A step of genomic DNA elimination using DNase I (QIAGEN) was included in the mRNA purification protocol. For each sample tested, 500 ng of mRNA was then reverse-transcribed using the First-strand cDNA synthesis kit from QIAGEN. Expression of 84 genes related to cell death was quantified using the RT^2^ Profiler PCR Array Cell Death Pathway Finder from SABIOSCIENCES-QIAGEN, which includes internal controls, such as housekeeping genes, reverse transcription control, positive PCR control and genomic DNA contamination control. SYBER Green fluorescence was detected using LightCycler 480 (ROCHE), and the crossing point (Cp) of PCR reactions were calculated using the Second Derivative Maximum analysis method. Data were analyzed using the online PCR Array Data Analysis Web Portal (www.SABiosciences.com/pcrarraydataanalysis.php), which allowed interpretation of control wells, validation of the experiments, normalization and quantification using the 2^−ΔCp^ method. 4 groups were studied: siSTAT3-and scramble-hMφ each either infected by H37Rv-GFP or non-infected. Expression of the 84 tested genes of the 4 groups was then normalized to that of the scramble group in absence of infection. The criteria used were fold change >2 or a p-value < 0.05. Six genes were found modulated by STAT3 in absence of infection and were then excluded from the analysis. Upon *M. tuberculosis* infection, 43 genes had their expression level modified in both siSTAT3 and scramble groups relative to scramble group in absence of infection and 16 genes were finally selected based on p-value < 0.05. Finally, 9 genes were specifically modulated by STAT3 upon infection.

### Detection of mRNA by RT-qPCR

After infection, macrophages were washed once with DPBS and mRNA was purified by using the RNeasy purification kit from QIAGEN, according to the manufacturer’s recommendations. For SOCS3 and NOS2 detection, at least 0.8 μg and 1.5 μg of mRNA was then reverse-transcribed using the Reverse Transcription System kit from PROMEGA, respectively. Relative expression of SOCS3 and NOS2 cDNA was detected using specific FAM-labeled Taqman Probe from Life Technologies which targeted human SOCS3 (Hs01000485) or human NOS2 (Hs01075529). We used GAPDH as a housekeeping gene. GAPDH was detected using FAM-labeled Taqman Probe from Life technologies targeting human GAPDH (Hs02758991). FAM fluorescence was detected using LightCycler 480 (ROCHE), and the crossing points (Cp) of PCR reactions were calculated using the Basic Relative Quantification method. Data were then normalized and quantified using the 2^−∆Cp^ method.

## Additional Information

**How to cite this article**: Queval, C. J. *et al*. STAT3 Represses Nitric Oxide Synthesis in Human Macrophages upon *Mycobacterium tuberculosis* Infection. *Sci. Rep.* **6**, 29297; doi: 10.1038/srep29297 (2016).

## Supplementary Material

Supplementary Table S1

Supplementary Table S2

Supplementary Information
