# Supplementary material for: STAT3 Represses Nitric Oxide Synthesis in Human Macrophages upon Mycobacterium tuberculosis Infection
Source: Sci Rep. 2016 Jul 7;6:29297. doi: 10.1038/srep29297 (PMC4935992; doi:10.1038/srep29297)
Supplement: Supplementary Information [file srep29297-s3.pdf]

STAT3 Represses Nitric Oxide Synthesis in Human Macrophages upon *Mycobacterium tuberculosis* Infection

Christophe J. Queval[1], Ok-Ryul Song[1], Nathalie Deboosère[1,] Vincent Delorme[1], Anne-Sophie Debrie[1], Raffaella Iantomasi[1], Romain Veyron-Churlet[1], Samuel Jouny[1], , Keely Redhage[1], Gaspard Deloison[1], Alain Baulard[1], Mathias Chamaillard[1], Camille Locht[1], Priscille Brodin[1]*

Supplementary Informations

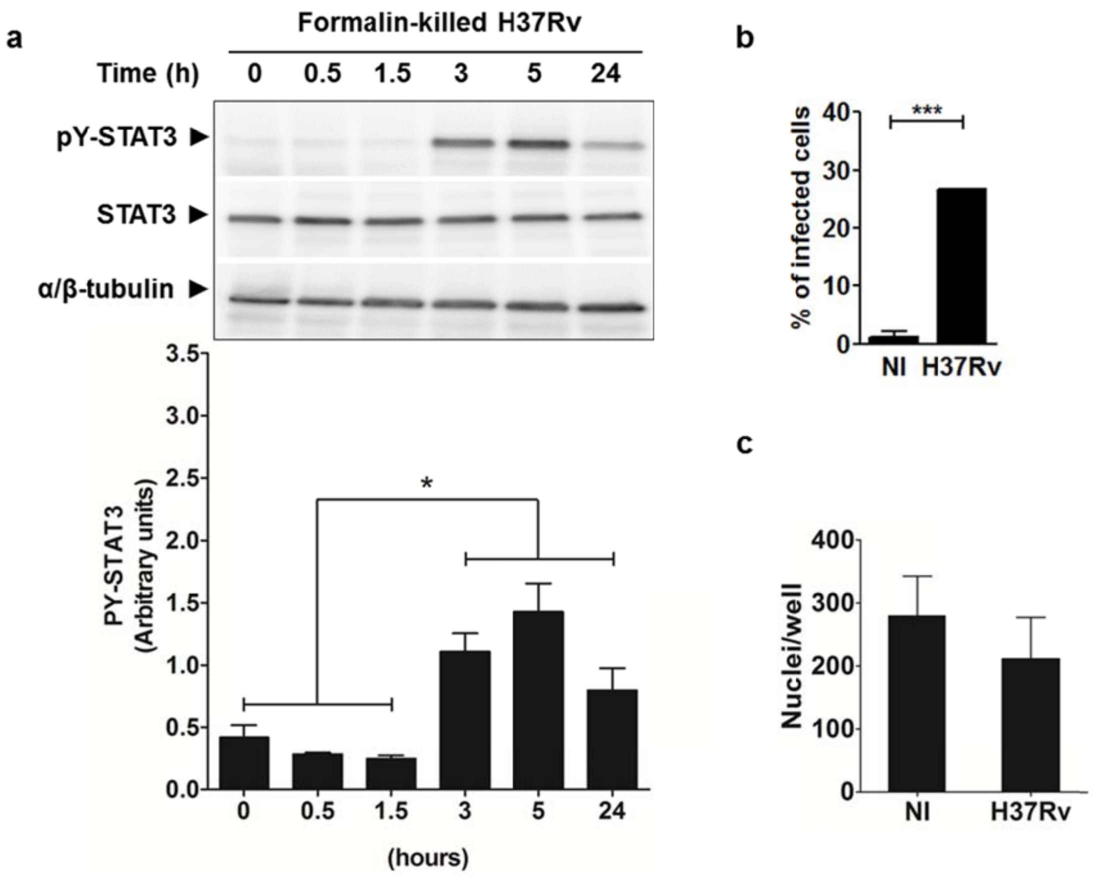

Supplementary Figure S1 (related to Figure 1): *M. tuberculosis* induces early activation of STAT3 signaling in both infected and bystander macrophages

(**a**) Kinetics of STAT3 activation in hMΦ macrophages infected with killed H37Rv at a MOI of 2. STAT3 activation was analyzed by immunoblotting using anti- PY^{705}-STAT3. Blotting with anti- α/β -tubulin was used to confirm gel loading. Immunoblots are representative of two independent experiments. Full-length blots are presented in Supplementary Figure S13. (**b, c**) Quantification of the percentage of infected cells (**b**) and cell viability (**c**) obtained for the samples used for quantification of STAT3 nuclear translocation. For each condition, uninfected hMΦ (NI) and H37Rv-GFP-infected macrophages (H37Rv) were analyzed in triplicates which correspond to approximately 900 cells. Values reported represent the mean percentage of infected macrophages (**b**) or cell viability (**c**) ±SD for two independent experiments with two different donors.

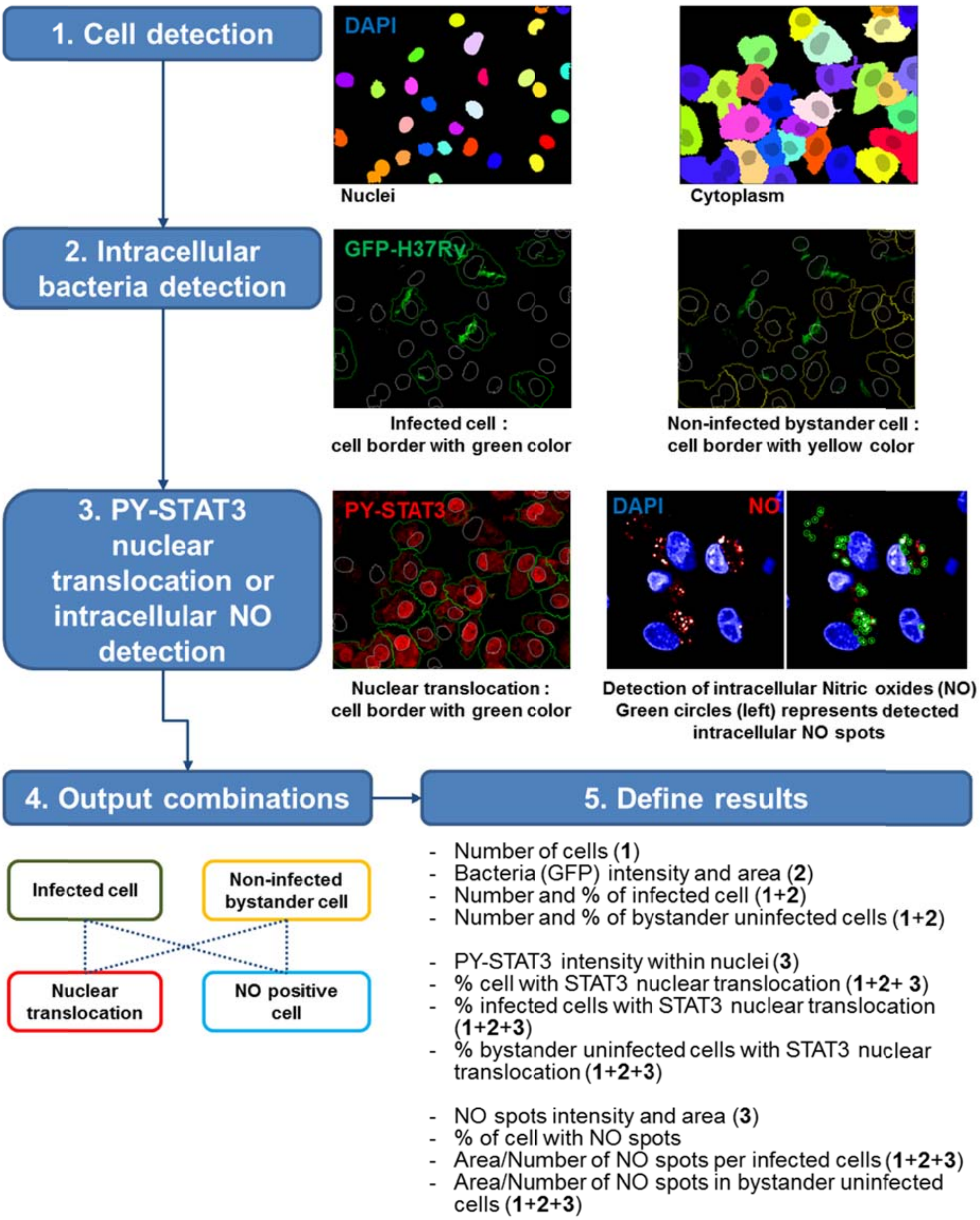

Supplementary Figure S2 (related to Figures 1, 4 and 5):
Scheme representing image-based analysis method performed with the image-analysis software Columbus 2.5.1 (PerkinElmer). **1**. Cell detection: cell nuclei and cell cytoplasm

were detected using DNA dye; DAPI (blue channel). **2**. Intracellular bacteria detection: data related to intensity and area of *M. tuberculosis*-GFP was measured in the green channel. **3**. STAT3 nuclear translocation and NO production were determined in the red channel. **4**. Outputs from 1, 2 and 3 were then combined to split the cell populations, infected and non-infected bystander cells, used to quantify the STAT3 nuclear translocation or NO production. **5**. List of values obtained from the different analysis or combination.

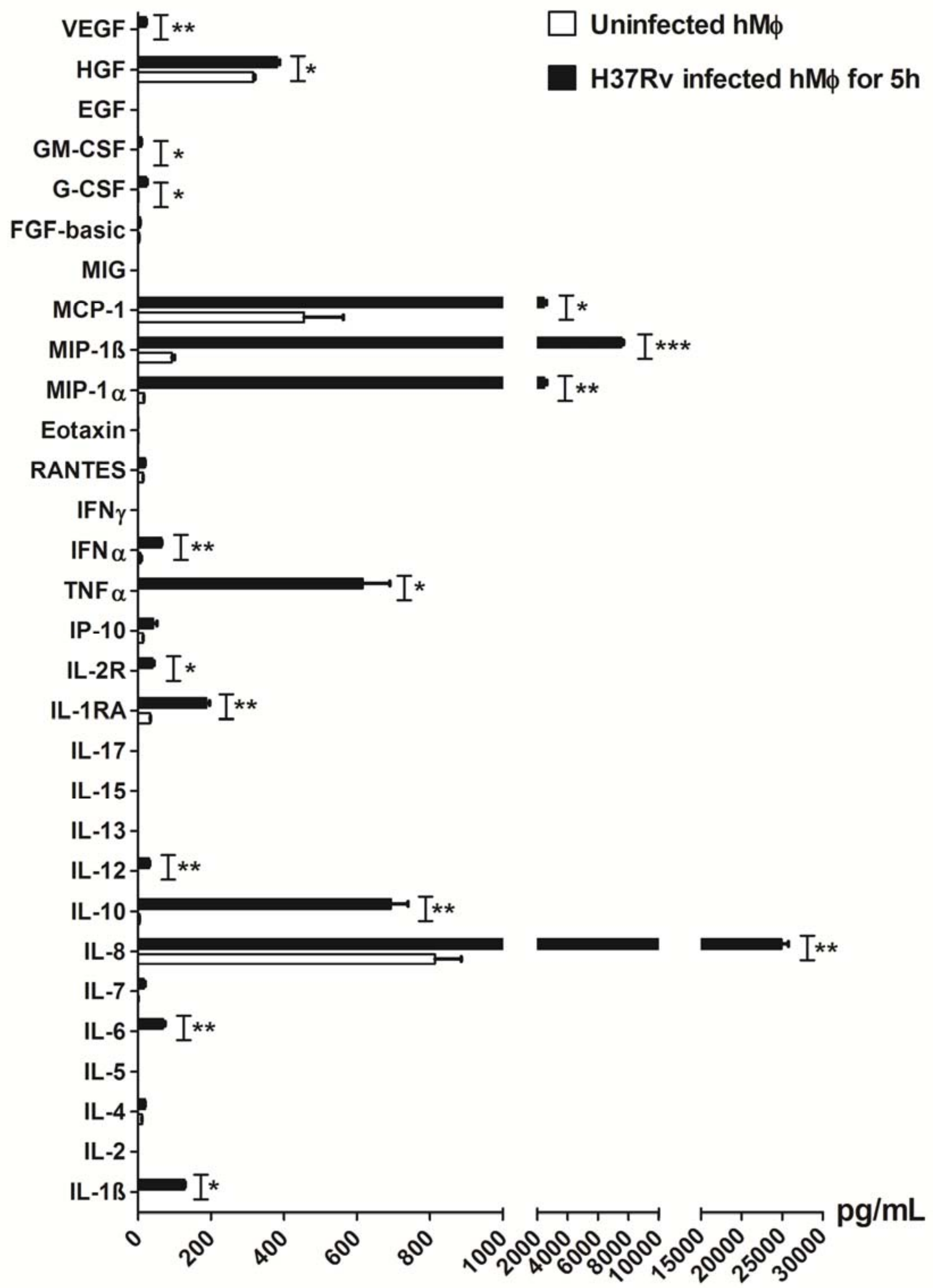

Supplementary Figure S3 (related to Figure 2): Early cytokine expression profile in H37Rv-infected macrophages

Cytokine release was quantified in supernatants collected from uninfected- hMΦ (white bars) or hMΦ infected with H37Rv-GFP for 5 hours at a MOI of 2 (black bars). A set of 30 cytokines was analyzed using Cytokine Human 30-Plex array (Life Technologies). Values represent average concentrations of cytokine release ±SEM, obtained from two independent donors, each tested in duplicate. Asterisks indicate the statistically significant differences between supernatants collected from uninfected cells and H37Rv-GFP-infected cells, calculated using a Student t-test. (* $p < 0.05$, ** $p < 0.01$, *** $p < 0.001$).

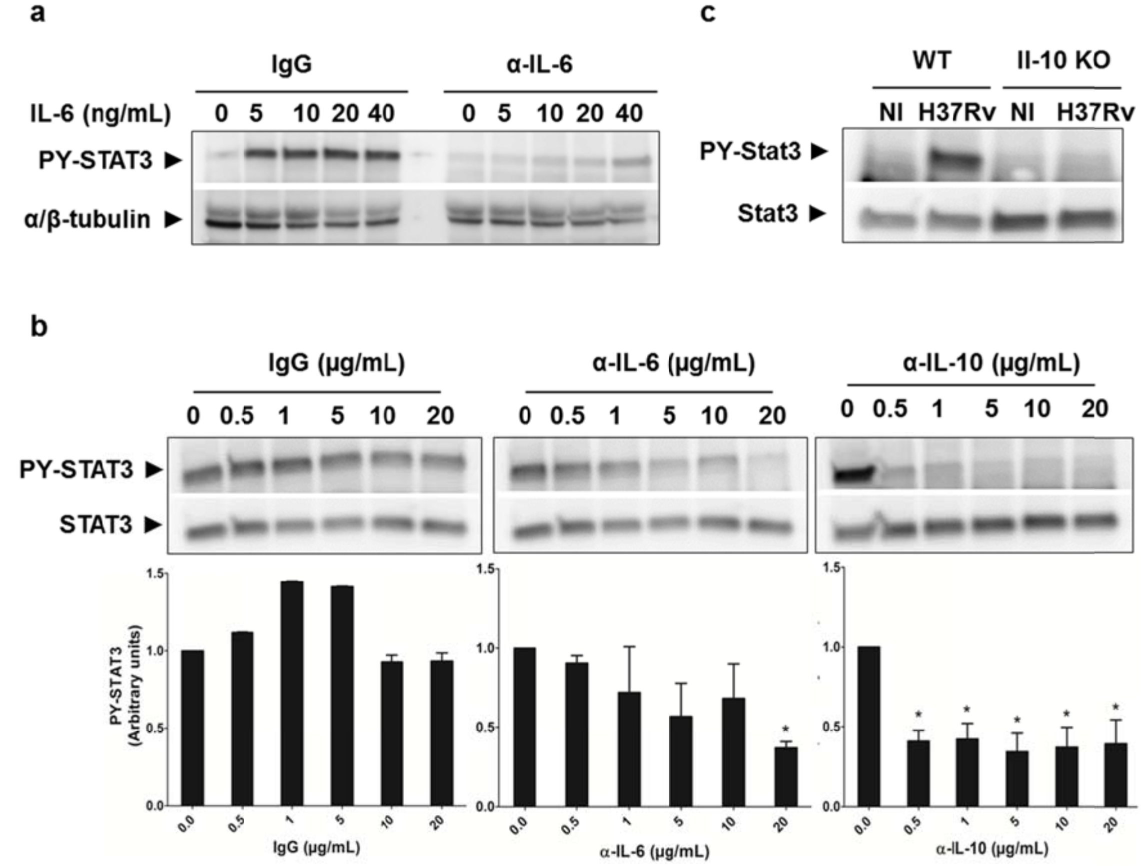

Supplementary Figure S4 (related to Figure 2): Activation of STAT3 mainly occurs through IL-10 signaling

(**a**) hMΦ were treated for 1 hour with various concentrations of IL-6 in presence of 10µg/mL of neutralizing anti-IL-6 (α-IL-6) or IgG as control antibody. (**b**) hMΦ were infected with H37Rv-GFP at a MOI of 2 for 3 h (H37Rv) in the presence of various concentrations (µg/mL) of control antibody (IgG Ctrl), neutralizing anti-IL-6 (α-IL-6), neutralizing anti-IL-10 (α-IL-10). Reported values represent the means of 2 independent experiments from two independent donors ±SEM and correspond to the relative STAT3 phosphorylation. Asterisks indicate the statistically significant differences, calculated using the Student t-test, between treated sample compare to untreated sample (0 µg/mL), (* $p<0.05$). (**c**) BMDM from Wild type (WT) or Il-10 KO mice were infected (H37Rv) or not (NI) with H37Rv-GFP at a MOI of 2 for 5 h (H37Rv). Immunoblot is representative of 3 independent experiments. For all immunoblots, STAT3 activation was analyzed by immunoblotting using anti-PY705-STAT3 antibody and anti-STAT3 or

anti-α/β-tubulin were used to confirm gel loading. Full-length blots are presented in Supplementary Figure S14.

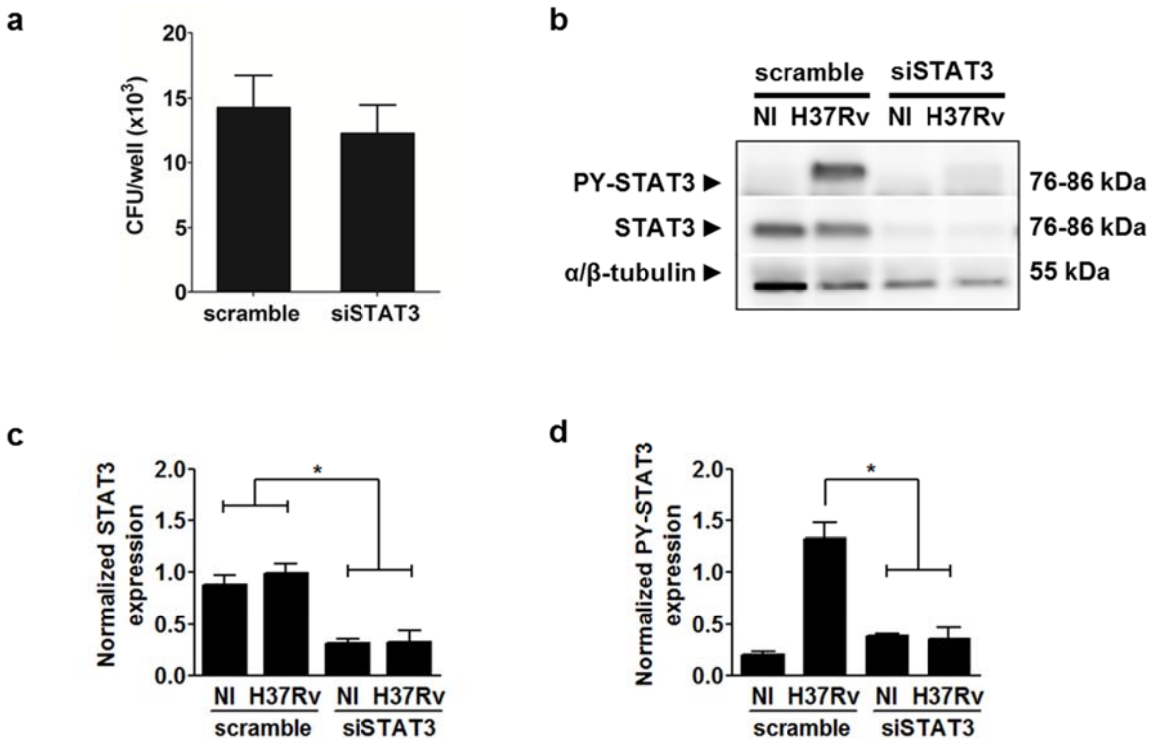

Supplementary Figure S5: STAT3 silencing in human macrophages

(**a**) siSTAT3 and scramble hMΦ were infected with H37Rv-GFP at a MOI of 0.5 for 4 hours. Infected cells were then lysed and the titer of intracellular H37Rv-GFP was determined by CFU counting. (**b**) Quantification of STAT3 silencing efficiency. siSTAT3 and scramble hMΦ were infected with H37Rv for 24h. Non-infected cells (NI) were used as negative controls. STAT3 expression (STAT3) and activation (PY-STAT3) were analyzed by immunoblotting using specific antibodies recognizing STAT3 and PY^{705}-STAT3, respectively. Probing with anti-α/β-tubulin was used to confirm gel loading. Full-length blots are presented in Supplementary Figure S15. (**c**) Quantification of the relative STAT3 expression in hMΦ silenced for STAT3. (**d**) Quantification of the relative STAT3 phosphorylation in hMΦ silenced for STAT3. Immunoblot is representative of three independent experiments, each of them performed with pooled macrophages from 2 different donors. Values reported on the graphics represent the mean of 3 experiments ±SEM. Asterisks indicate the statistically significant differences between compared conditions, calculated using the Student t-test (* $p < 0.05$).

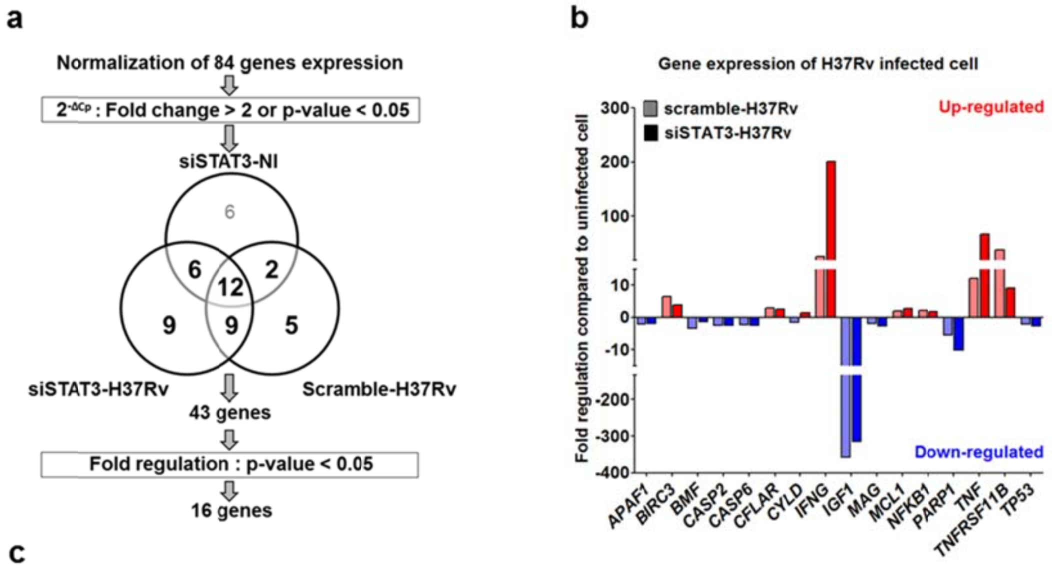

c

Gene expression of siSTAT3 hMΦ relative to scramble hMΦ upon *M.tuberculosis* infection		
Gene symbol	Fold regulation	P-value
BMF	2.5567	0.0007
CYLD	2.0153	0.0056
IFNG	7.5386	0.0072
MCL1	1.3419	0.0202
TNF	5.4678	0.0007
BIRC3	-1.6924	0.0202
MAG	-1.4003	0.0246
PARP1	-1.8996	0.0044
TNFRSF11B	-4.2941	0.0014

Supplementary Figure S6 :STAT3 modulates various cellular pathways during *M. tuberculosis* macrophage infection
siSTAT3 and scramble hMΦ were infected with H37Rv-GFP at a MOI of 1 for 24 h. The expression of 84 genes, mainly involved in cell death pathways, was measured by RT-qPCR using RT^2 Profiler Cell Death Pathway Finder. All values correspond to the averages of three experiments, each of them were performed with pooled hMΦ from 2 different donors. (**a**) Graphics representing the selection of hMΦ genes modulated by STAT3 during *M. tuberculosis* infection (detailed in Materials and Methods, “mRNA purification and data analysis from RT-qPCR analysis”). (**b**) Expression of 16 genes differentially modulated in infected-hMφ (scramble-H37Rv) and infected- hMΦ silenced for STAT3 (siSTAT3-H37Rv). Values correspond to the fold change of gene expression compared to the control group (scramble-NI), for which p <0.05. (**c**) Differences of gene fold regulation between siSTAT3-H37Rv and scramble-H37Rv groups.

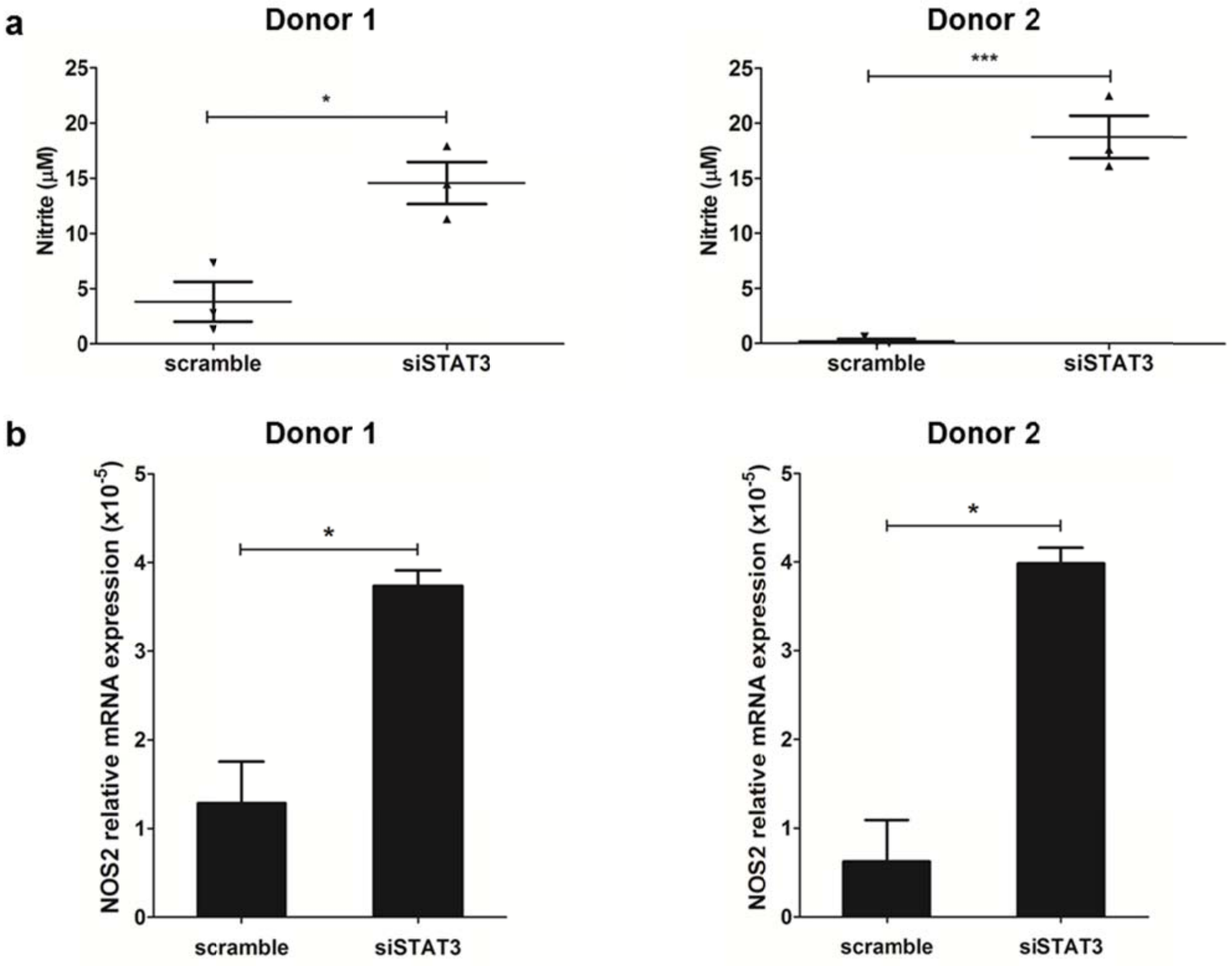

Supplementary Figure S7 (related to Figure 4): STAT3 silencing in human macrophage enhances Nitric oxides production in response to *M. tuberculosis* infection.

(**a, b**) hMΦ were transfected with scramble control or STAT3 siRNA and were then infected with H37Rv-GFP at MOI of 2. (**a**) Quantification of Nitrite (μM) in H37Rv-infected hMΦ-scramble supernatants using Greiss detection reagents. (**b**) scramble-hMΦ and siSTAT3-hMΦ were infected with H37Rv. After 24 hours of infection, mRNA were extracted and NOS2 mRNA expression was analyzed by RT-qPCR. The relative NOS2 mRNA expression was analyzed with hMΦ from same donors tested in (**a**).

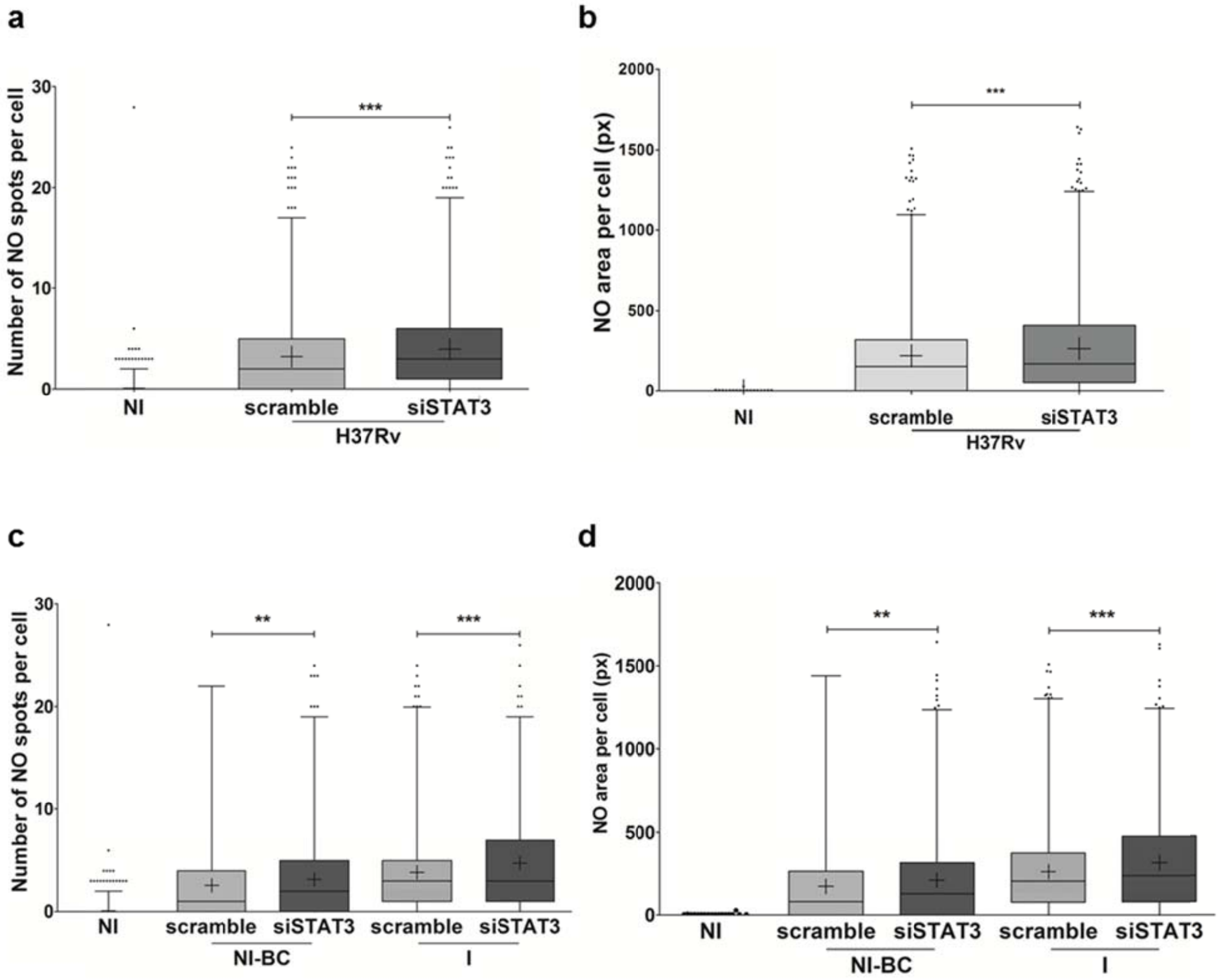

Supplementary Figure S8 (related to Figure 4): STAT3 signaling prevents nitric oxide production in both *M. tuberculosis*-infected and bystander uninfected macrophages

(**a, b**) Image-based quantification of NO spots (pixels) per cell (**a**) and NO area (pixels) per cell (**b**) in uninfected hMΦ (NI), and H37Rv-GFP (H37Rv) infected-hMΦ control (scramble) or silenced for STAT3 (siSTAT3). (**c**, **d**) Image-based quantification of NO spots (pixels) per cell (**c**) and NO area (pixels) per cell (**d**) within H37Rv-GFP infected-hMΦ control (scramble) or silenced for STAT3 (siSTAT3) with intracellular H37Rv (I) or non-infected bystander hMΦ (NI-BC). Data represent the average of 2 independent experiments using pooled macrophages from two independent donors, each tested in triplicate. Asterisks indicate the statistically significant differences between compared conditions, calculated using the Mann-Whitney test (** $p<0.01$, *** $p<0.001$).

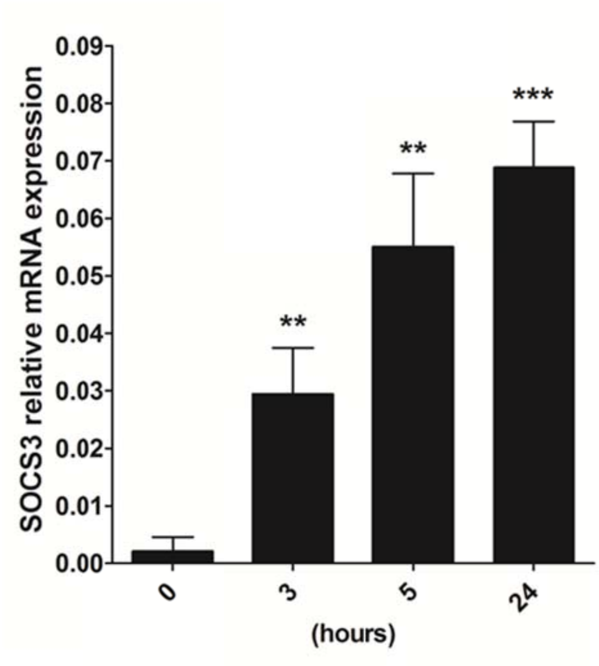

Supplementary Figure S9: Kinetics of SOCS3 expression in human macrophage infected with H37Rv.

hMΦ were infected with H37Rv at MOI of 2. mRNA were extracted at different time of infection and SOCS3 mRNA expression was analyzed by RT-qPCR. The relative SOCS3 mRNA expression is representative of two independent experiments performed with two different donors. Values represent the SOCS3 relative mRNA expression ±SD. Asterisks indicate the statistically significant differences compared to T0, calculated using the Student t-test (** $p < 0.01$, *** $p < 0.001$)

a

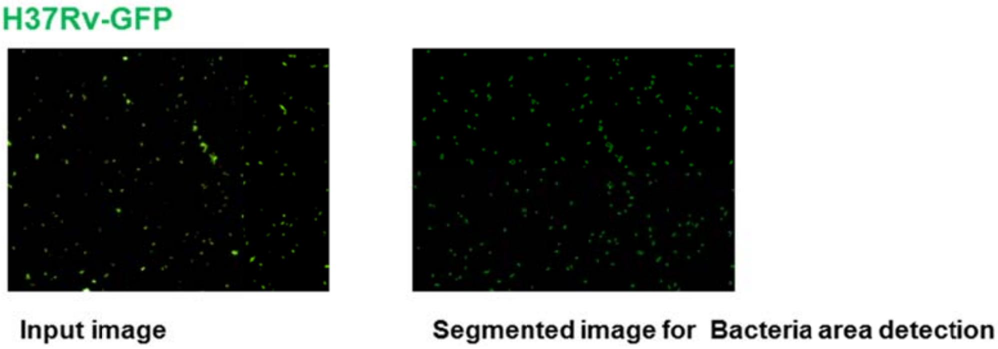

b

Input Image	Stack Processing : Individual Planes<br>Flatfield Correction : None		

M. tuberculosis-GFP (H37Rv-GFP) detection

Find Spots	**Channel :** Exp2Cam2<br>**ROI :** Whole Image	**Method :** B<br>Detection Sensitivity : 0.5<br>Splitting Coefficient : 0.5<br>Calculate Spot Properties	Output Population : Spots
Calculate Intensity Properties	**Channel :** Exp2Cam2<br>**Population :** Spots	**Method :** Standard<br>Mean	Output Properties : Intensity Spot Exp2Cam2
Select Population	**Population :** Spots	**Method :** Filter by Property<br>Intensity Spot Exp2Cam2 Mean : > 60	Output Population : Bacteria
Define Results	**Method :** List of Outputs<br>**Population : Spots**<br>Number of Objects<br><br>**Population : Whole Image**<br><br>**Population : Bacteria**<br>Number of Objects<br>Spot Area [px²] : Sum		Output Population : Total Bacteria Area

c

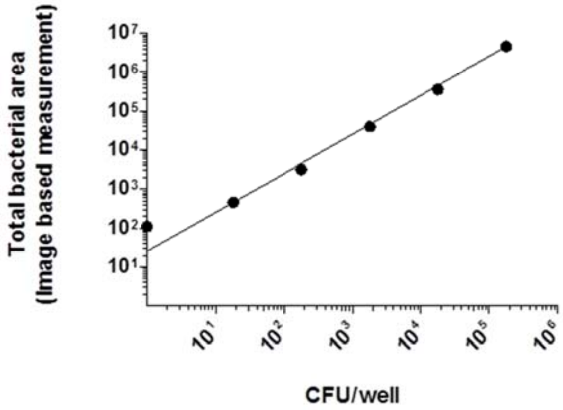

Supplementary Figure S10 (related to Figure 5) Image analysis of bacteria area:

(**a, b**) Representative confocal image of H37Rv-GFP bacteria (left) and corresponding bacteria area segmentation (right) using built-in Columbus script method B with on an Intensity Spot Threshold of 60 (**c**) Graphic representing the correlation of fluorescent H37Rv-GFP area per well (y-axis) in function of the number of bacteria harvested from axenic culture expressed in CFU per well (x-axis). ($R^2 = 0.9883$)

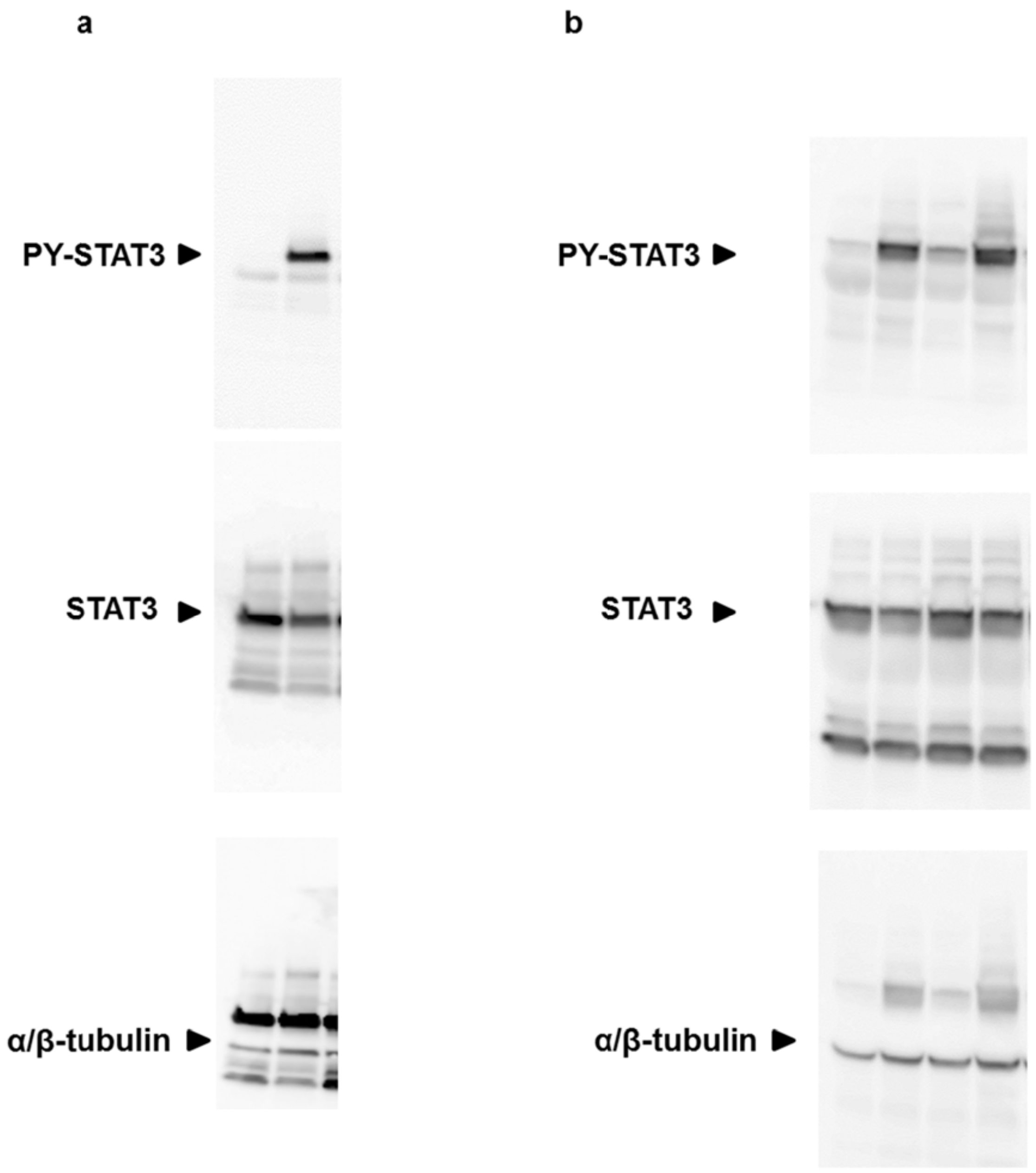

Supplementary Figure S11. Full-length blots corresponding to Figure 1

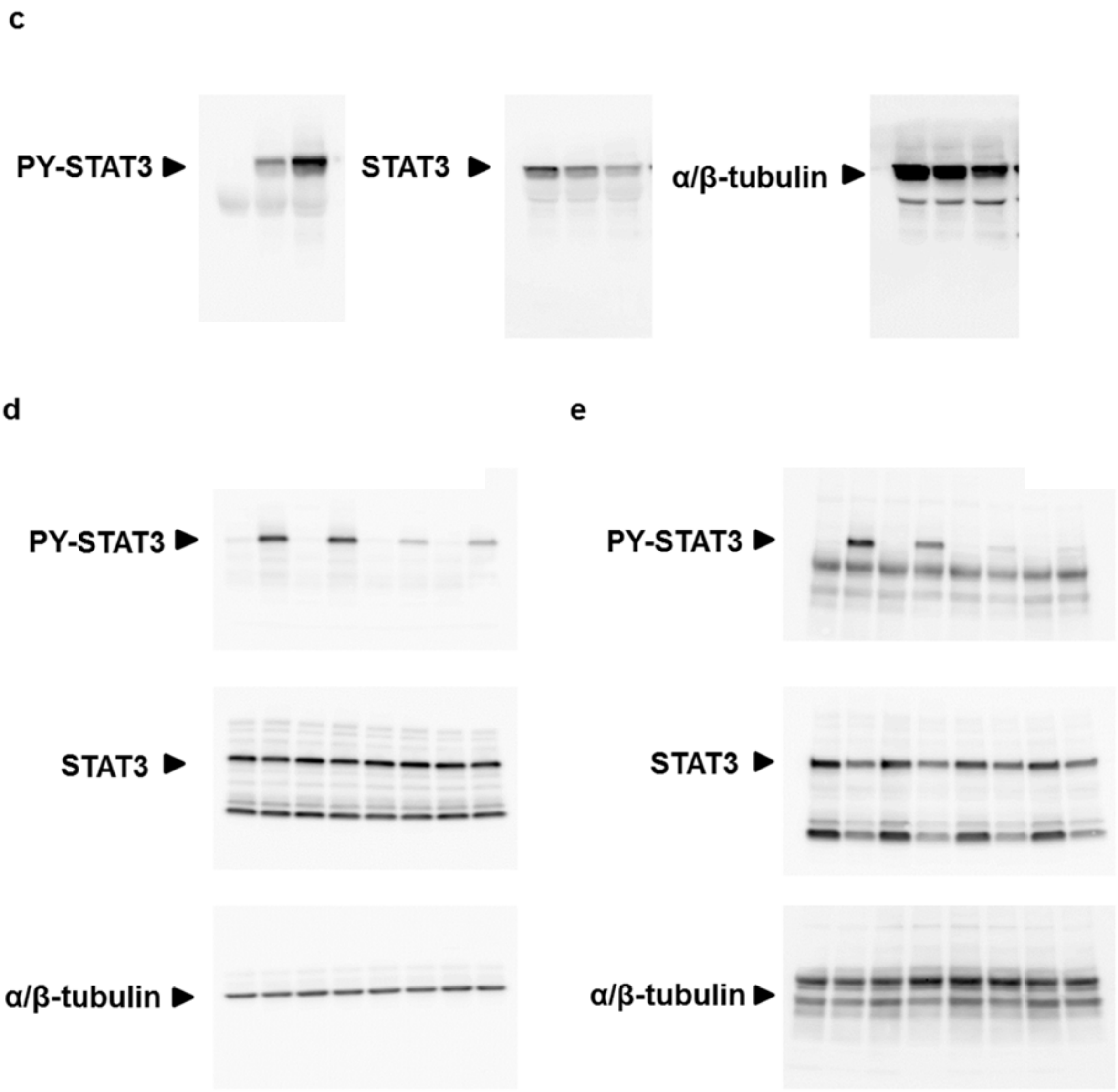

Supplementary Figure S12. Full-length blots corresponding to Figure 2

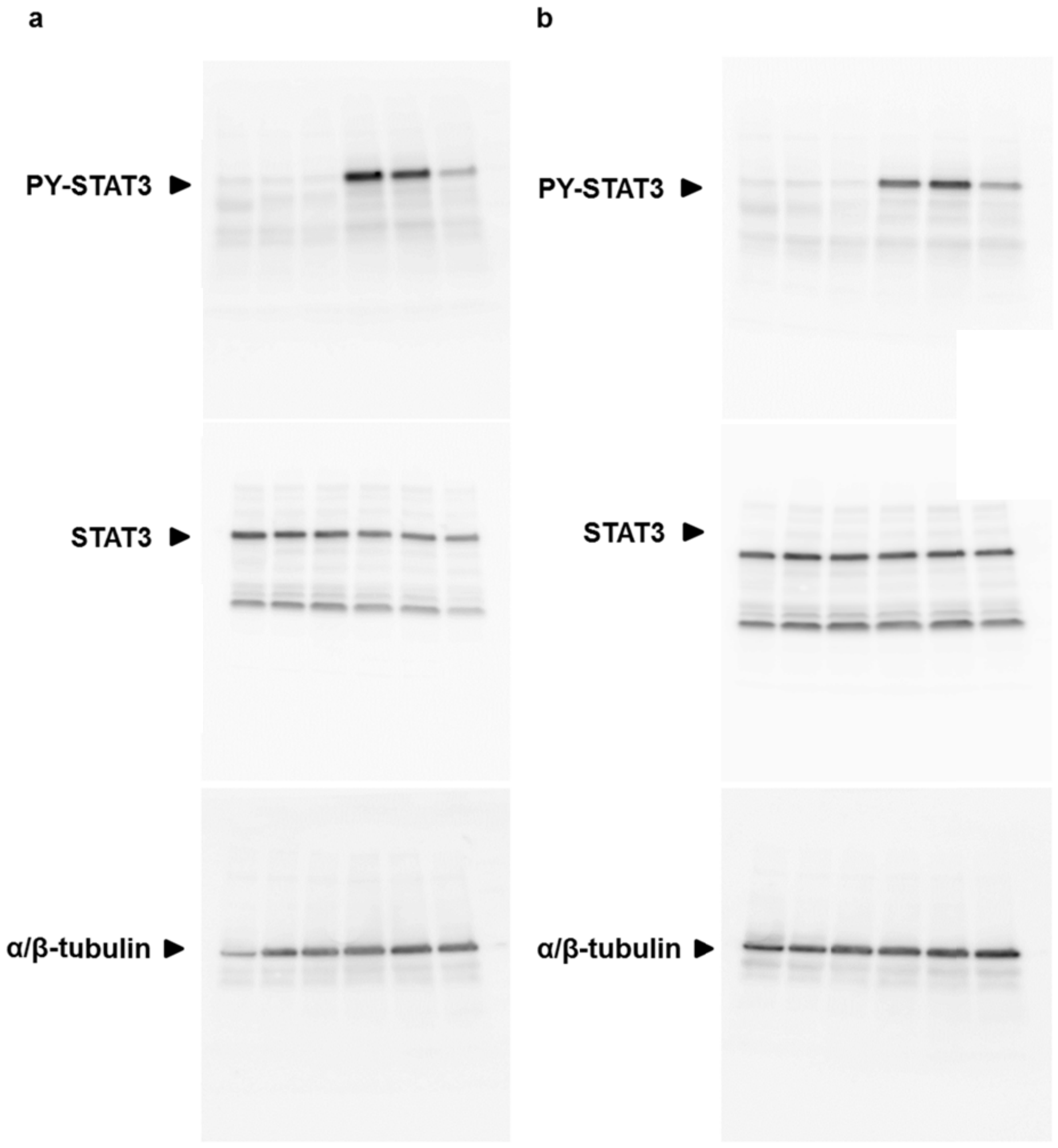

Supplementary Figure S13. Full-length blots corresponding to Figure S1

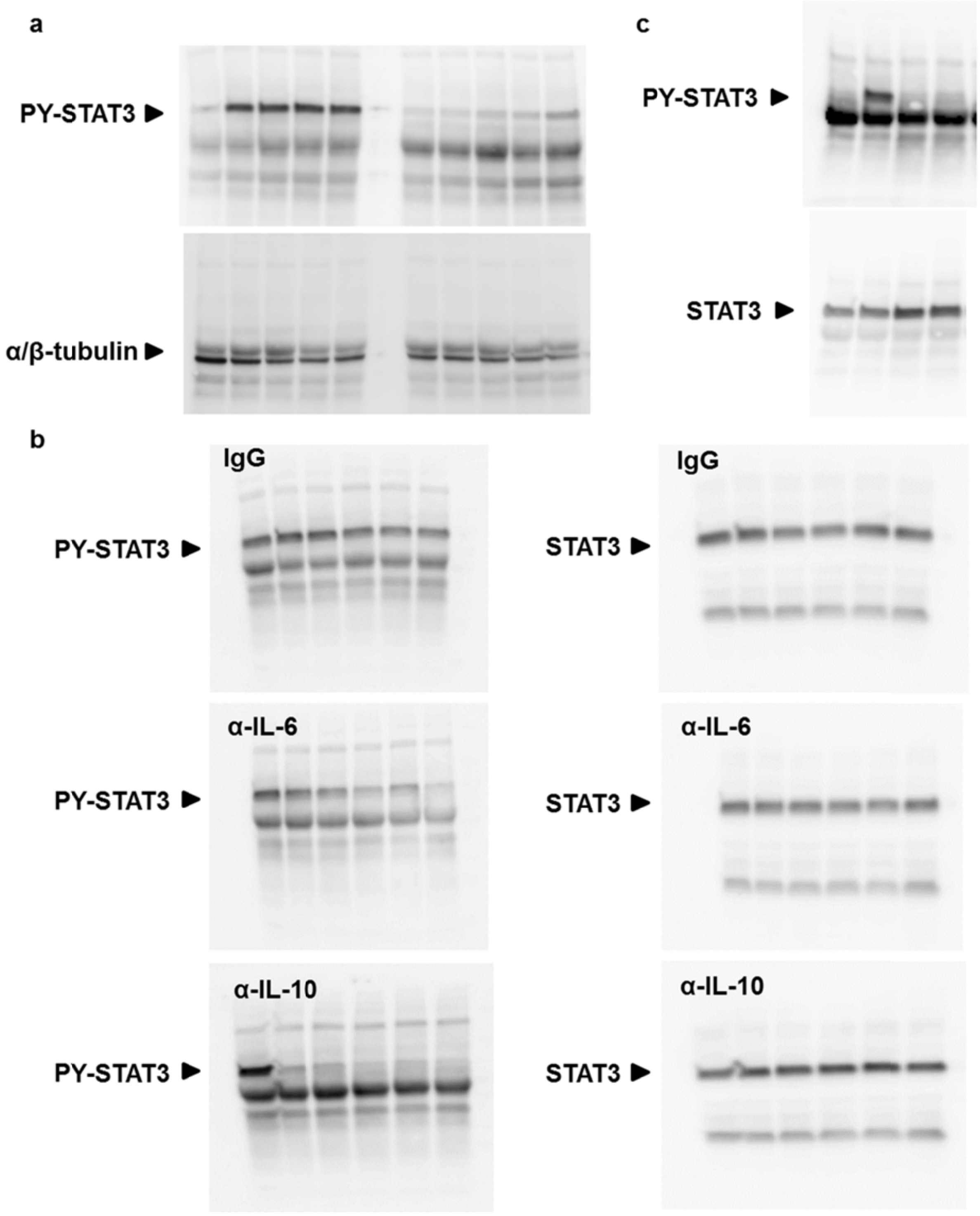

Supplementary Figure S14. Blot corresponding to Figure S4

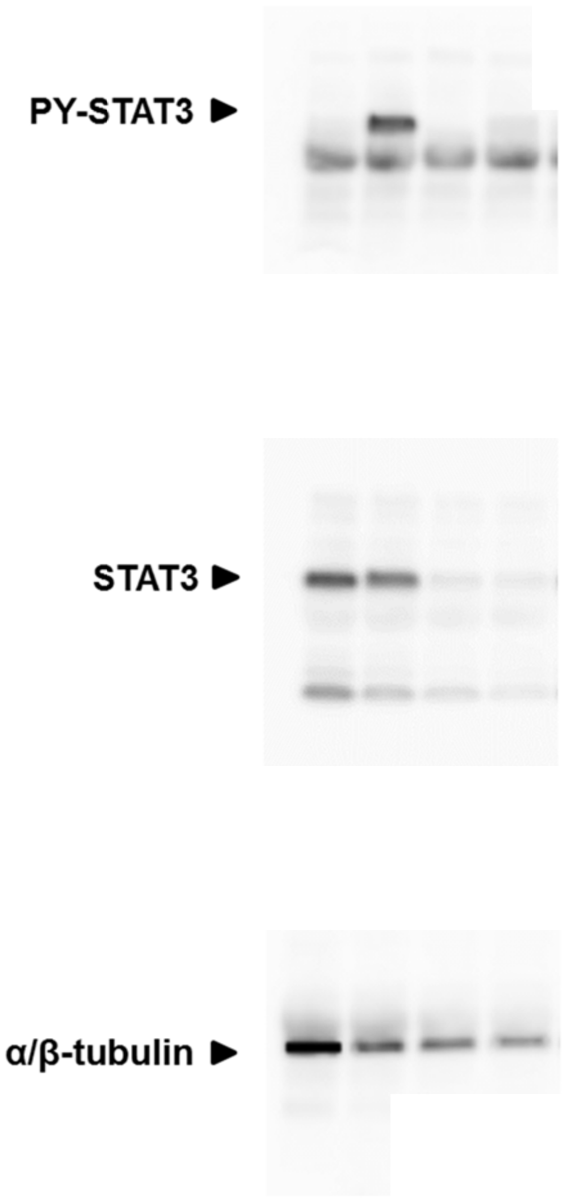

Supplementary Figure S15. Full-length blots corresponding to Figure S5b

Supplementary Table S1: Table S1-1, Columbus Script for Fig 1b, **Table S1-2,** Columbus Script for Fig 4b, **Table S1-3,** Acapella Script for Fig 5b

Supplementary Table S2: Table S2-1, Cell-death Pathway Finder, **Table S2-2,** List of genes significantly modulated in both scramble-H37Rv and siSTAT3-H37Rv groups, **Table S2-3,** Variation of cytokine profile in human primary macrophages infected with H37Rv for 24h, **Table S2-4,** Variation of cytokine profile in H37Rv-human primary macrophages silenced for STAT3
